# Conditional depletion of macrophages ameliorates cholestatic liver injury and fibrosis via lncRNA-H19

**DOI:** 10.1038/s41419-021-03931-1

**Published:** 2021-06-24

**Authors:** Xinbei Tian, Ying Wang, Ying Lu, Weipeng Wang, Jun Du, Shanshan Chen, Huiping Zhou, Wei Cai, Yongtao Xiao

**Affiliations:** 1 Division of Pediatric Gastroenterology and Nutrition, Xin Hua Hospital, School of Medicine, Shanghai Jiao Tong University, Shanghai, China; 2 Shanghai Key Laboratory of Pediatric Gastroenterology and Nutrition, Shanghai, China; 3 Shanghai Institute of Pediatric Research, Shanghai, China; 4 Department of Pediatric Surgery, Xin Hua Hospital, School of Medicine, Shanghai Jiao Tong University, Shanghai, China; 5 Department of Microbiology and Immunology and McGuire Veterans AfSfairs Medical Center, Virginia Commonwealth University, Richmond, VA USA

**Keywords:** Translational immunology, Cholestasis

## Abstract

Although macrophages are recognized as important players in the pathogenesis of chronic liver diseases, their roles in cholestatic liver fibrosis remain incompletely understood. We previously reported that long noncoding RNA-H19 (lncRNA-H19) contributes to cholangiocyte proliferation and cholestatic liver fibrosis of biliary atresia (BA). We here show that monocyte/macrophage CD11B mRNA levels are increased significantly in livers of BA patients and positively correlated with the progression of liver inflammation and fibrosis. The macrophages increasingly infiltrate and accumulate in the fibrotic niche and peribiliary areas in livers of BA patients. Selective depletion of macrophages using the transgenic CD11b-diphtheria toxin receptor (CD11b-DTR) mice halts bile duct ligation (BDL)-induced progression of liver damage and fibrosis. Meanwhile, macrophage depletion significantly reduces the BDL-induced hepatic lncRNA-H19. Overexpression of H19 in livers using adeno-associated virus serotype 9 (AAV9) counteracts the effects of macrophage depletion on liver fibrosis and cholangiocyte proliferation. Additionally, both H19 knockout (H19^−/−^) and conditional deletion of H19 in macrophage (H19^ΔCD11B^) significantly depress the macrophage polarization and recruitment. lncRNA-H19 overexpressed in THP-1 macrophages enhance expression of Rho-GTPase CDC42 and RhoA. In conclusions, selectively depletion of macrophages suppresses cholestatic liver injuries and fibrosis via the lncRNA-H19 and represents a potential therapeutic strategy for rapid liver fibrosis in BA patients.

## Introduction

Cholestatic liver diseases, such as biliary atresia (BA), usually lead to end-stage liver diseases [1, 2]. BA is a severe liver disease in neonates featuring cholestasis and severe liver fibrosis [3, 4]. Due to the poor understanding of its pathogenesis, BA clinical management remains challenging other than a liver transplant. It is reported that the infiltration of inflammatory macrophages increased in BA patients via (MCP)-1/(C-C motif) ligand 2 (CCL2) and its receptor C-C chemokine receptor 2 (CCR2) and promoted cholangiocyte injury and disease progression [5]. Hepatic macrophages include tissue-resident kupffer cells (KCs) and macrophages recruited from the circulating bone marrow-derived monocyte lineage. KCs are originated from fetal precursor cells and located within the periportal area of hepatic sinusoids [6]. The liver macrophages derived from monocytes are recruited from circulation mainly through the CCL2/CCR2 pathway [7]. Previous studies also demonstrated that the deficiency of either Ccl2 or Ccr2 could largely alleviate cholestatic liver injury in mice [8, 9]. However, the roles of macrophages in liver fibrosis are seemingly ambivalent [10, 11]. In the current study, we used transgenic CD11b-DTR mice to examine the role of macrophages in cholestatic liver fibrosis. Mice are not sensitive to diphtheria toxin (DT) as the murine DTR does not bind to DT. Macrophages in CD11b-DTR mice specifically express the human DTR and can be depleted by a simple intravenous injection of DT [12, 13]. CD11b-DTR mice have been shown to have inflammatory macrophages that can be specifically depleted from solid organs in vivo [12].

The previous studies reported that long noncoding RNA (lncRNA) H19, an imprinted and maternally expressed gene, was significantly induced in the cholestatic liver [14]. H19 not only contributes to bile acid dysregulation via inhibiting small heterodimeric partner expression in hepatocytes [15] but also promotes liver fibrosis by activating hepatic stellate cells [16]. We also reported that hepatic and serum exosomal H19 levels are positively correlated with the severity of fibrotic liver injuries in BA patients. H19 deficiency protects the mice from bile duct ligation (BDL)-induced cholangiocyte proliferation and liver fibrosis via inhibiting bile acid-induced expression and activation of sphingosine 1-phosphate receptor 2 (S1PR2) [17]. We also showed that the expression level of H19 in the liver macrophage increased in BA patients. However, the contribution of macrophages to cholestatic liver injury in BA patients and the potential mechanisms remain to be fully elucidated.

## Materials and methods

### Human liver specimens

A total of 44 liver specimens were retrieved from BA patients undergoing surgery. Twelve normal adjacent non-tumor tissues taken from hepatoblastoma patients were used as healthy controls (HC). All patients’ guardians provided written informed consent. The patients’ characteristics are presented in Table S1. This study was approved by the Faculty of Medicine’s Ethics Committee of Xin Hua Hospital (XHEC-D-2020-187). All methods in this study were carried out following the relevant guidelines.

### Animal studies

CD11b-DTR mice (Fig. S1A) and H19^−/−^ mice (ΔExon 1–5, Fig. S1B) with C57/BL6 background were obtained from Nanjing Biomedical Research Institute of Nanjing University (Nanjing, China). A total of 63 CD11b-DTR (male, *n* = 42; female, *n* = 21, approximately 7 weeks old) mice weighing 18–22 g were used in this study. All mice were subjected to either BDL or sham operation, as described previously [17]. The 2 weeks after the operation, sham mice and BDL mice were intraperitoneally (i.p.) injected with either (a) DT (25 ng/g) or (b) saline as a control every 3 days. Tissues and serum were harvested 24 h following the final injection and processed for quantitative reverse transcriptase PCR (qRT-PCR), western blotting analysis, histology, and immunohistochemistry analysis. WT and H19^−/−^ mice were subjected to BDL or sham operation as described above (*n* = 6–10). *H19*-floxed mice (Fig. S2A) were generated by using the CRISPR/Cas9 system in which exon 1–5 of murine *H19* was flanked with two loxp sequences. H19-floxed alleles (H19^fl/fl^) were bred with CD11B^CreERT2^ mice (Fig. S2B, GemPharmatech, Nanjing, China) to generated H19^fl/fl^: CD11B^CreERT2^ mice. The H19^fl/fl^: CD11B^CreERT2^ mice were subjected to either BDL or sham operation for 8 days. To deplete the H19 in macrophages in vivo (H19^**ΔCD11B**^), the H19^fl/fl^: CD11B^CreERT2^ mice were given tamoxifen via i.p injection at a dose of 75 mg/kg body weight daily for a total of three consecutive days. For overexpressing H19 in the livers of mice, the murine H19 (NR_130973) cDNA was subcloned into the adeno-associated virus serotype 9 (AAV9) transfer plasmid (Fig. S3) and transfected into HEK293T cells to produce AAV9-H19 vector genomes (Genechem, Shanghai, China). A dose of 1.0 × 10^11^ vector genomes of the AAV9-H19 or AAV9 controls were injected into CD11b-DTR mice via the tail vein. Seven days post-injection, the mice were subjected to the BDL operation and randomly divided into four groups (each group, *n* = 4–6): saline + Control AAV9 vector (AAV9-CTL), saline + AAV9-H19 (AAV9-H19), DT + Control AAV9 (AAV9-CTL + DT), and DT + AAV9-H19 (AAV9-H19 + DT). Mice were injected with either DT (20 ng/g, i.p.) or saline (i.p.) every 2 days. After 10-day BDL, liver tissues and serum were harvested for further research. All experimental protocols were approved by the Shanghai Jiao Tong University School of Medicine affiliated Xin Hua hospital Animal Care and Use Committee (XHEC-F-2020-009).

### Statistical analysis

All data are reported as the means ± SD. For comparisons of different groups, statistical significance was determined based on Student’s *t*-test or ANOVA analysis with Bonferroni correction. The relationships between two factors were tested with two-tailed Pearson’s correlations. *P* values <0.05 were considered to be statistically significant.

## Results

### The increased macrophages in BA livers positively correlate with progression of the inflammation, fibrosis, and angiogenesis

**Fig. 1:**
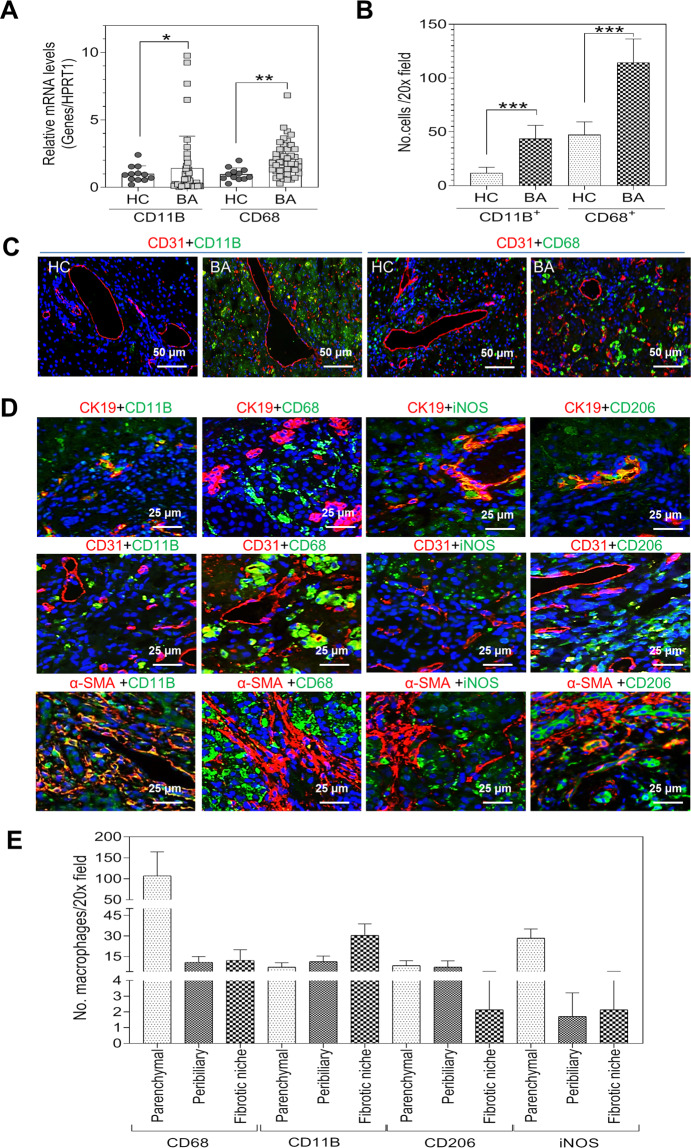
Increased macrophages accumulate in fibrotic niche and peribiliary areas of BA patients’ livers. **A** Relative mRNA levels of CD11B and CD68 in livers of BA patients (*n* = 44) and controls (*n* = 12) were determined by real-time PCR (RT-PCR). HPRT1 was used as an internal control. **B** Quantification of CD68^+^ and CD11B^+^ cells in liver sections of HC (*n* = 3) and BA (*n* = 5) by ImageJ. **C** Representative images of co-immunofluorescence of CD31 and CD68, CD31 and CD11B in livers of BA and HC. Scale bars: 50 μm. **D** Representative IF images of CK19, CD31, α-SMA, CD68, CD11B, CD206, and iNOS in BA patients (*n* = 6). Scale bars: 25 μm. **E** Graph of CD68, CD11B, CD206, and iNOS^+^ macrophages in BA patients were determined by ImageJ. Data were expressed as mean ± SD. Statistical significance relative to controls: *P < 0.05; **P < 0.01; ***P < 0.001. BA biliary atresia, HC healthy controls, HPRT1 hypoxanthine phosphoribosyl transferase 1.

As shown in Fig. 1A, the RT-PCR analysis showed that CD11B and CD68 mRNA levels were much higher in BA patients than in control subjects (Fig. 1A). Immunofluorescence (IF) staining showed that the infiltration of CD11B^+^ monocytes/macrophages and the number of CD68^+^ macrophages increased in livers of BA patients (Fig. 1B, C). The CD68^+^ macrophages mainly accumulated in the parenchymal area (Fig. 1D, E). The CD11B^+^ cells infiltrated and accumulated in the peribiliary areas and the fibrotic niche. Interestingly, both pro-inflammatory (iNOS^+^) and anti-inflammatory (CD206^+^) macrophages were observed in the parenchyma, the peribiliary areas, and around the fibrotic niche (Fig. 1D, E). The transmission electron microscope (TEM) analysis also showed the macrophages presented in the fibrotic niche and hepatic vascule (Fig. S4). RT-PCR analysis also showed that the mRNA levels of C–X–C motif chemokine ligand 3 (CXCL3), monocyte chemoattractant protein-1 (MCP-1, CCL2) and its receptor chemokine (C-C motif) receptor 2 (CCR2) were higher in livers of BA patients than in controls, but not significant (Fig. S5). The pro-inflammatory markers, chemokine (C-C motif) ligand 20 (CCL20), interleukin 6 (IL6), and C–X–C motif chemokine ligand 8 (CXCL8, IL8), were increased significantly in livers of BA patients relative to controls (Fig. S5). The marker genes for cholangiocyte proliferation and liver fibrosis, including keratin 19 (KRT19), actin, alpha 2 smooth muscle (ACTA2), collagen type I alpha 1 (COL1A1), and cystic fibrosis transmembrane conductance regulator (CFTR), were increased markedly in livers of BA patients compared to controls (Fig. S5). The mRNA expression levels of angiogenesis genes, including intercellular adhesion molecule-1 (ICAM1), platelet and endothelial cell adhesion molecule-1 (PECAM), C-type lectin domain family 4 member G (CLEC4G), and alanyl aminopeptidase and membrane (ANPEP), were also increased in the livers of BA patients compared to controls, but did not reach significance (Fig. S5). The linear regression analysis showed that the relative mRNA level of hepatic CD11B was correlated with CD68, CCL2, CCR2, and NOS2 (Fig. S6), as well as the pro-inflammatory markers, including IL1B, TNFA, and IL6, but there was no significant correlation between relative mRNA level of hepatic CD11B and IL10 (Fig. S6). In addition, hepatic CD11B mRNA level was positively correlated with mRNA levels of KRT19, ACTA2, COL1A1, CFTR, and angiogenesis marker genes, including ICAM1, PECAM, CLEC4G, and ANPEP (Fig. S6).

### Administration of DT selectively depleting macrophages ameliorates cholestatic liver injuries

**Fig. 2:**
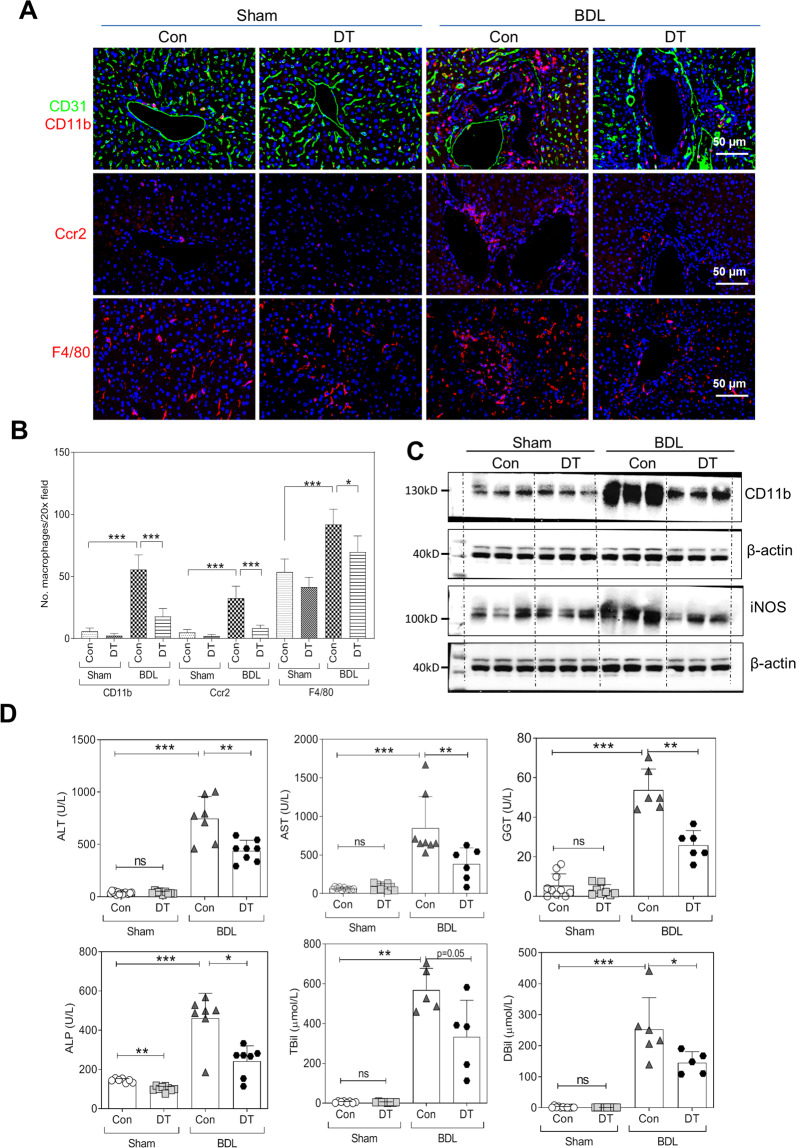
Diphtheria toxin (DT) injection depleted macrophages in the cholestatic livers and ameliorates liver injury. **A** The mice were divided into groups of Sham, Sham + DT, BDL, and BDL + DT (*n* = 5–8). Representative images of immunofluorescence (IF) staining for CD11b, Ccr2, F4/80, and CD31 in the livers of Sham, Sham + DT, BDL, and BDL + DT mice Scale bars: 50 μm. **B** Quantification of Ccr2^+^, F4/80^+^, and CD11b^+^ cells in panel **A** by ImageJ. **C** Western blotting analysis for CD11b and iNOS in Sham, Sham + DT, BDL, and BDL + DT mice. Representative images of three duplicate samples of the immune blottings are shown. **D** The levels of alanine aminotransferase (ALT), aspartate aminotransferase (AST), alkaline phosphatase (ALP), γ-glutamyl transferas (GGT), total bilirubin (TBil), and direct bilirubin (DBil) in serum (*n* = 5–13, each group). Data were expressed as mean ± SD. *P < 0.05; **P < 0.01; ***P < 0.001.

In order to determine the role of macrophages in cholestatic liver fibrosis, we transiently depleted macrophages using the CD11b-DTR transgenic mice treated with DT in a BDL mouse cholestatic model. A CD11b-DTR transgenic mouse is a well-established model that has the advantage of selectively depleting most tissue macrophages independently of their phagocytic activity [12, 13, 18, 19]. As shown in Fig. 2, IF staining showed that the percent positivity for CD11b^+^ monocytes/macrophages, F4/80^+^ macrophages, as well as Ccr2^+^ macrophages increased significantly and accumulated in the livers of BDL mice, which were depleted after DT administration (Fig. 2A, B). The administration of DT to wild-type (WT) control mice had no effect on macrophages infiltrating into the cholestatic livers after 2 weeks’ BDL (Fig. S8A,B). The RT-PCR analysis indicated that BDL-induced increase of hepatic levels of CD11b, Ccr2, F4/80, CD68, CD206, and Nos2 were significantly reduced by DT treatment (Fig. S7B). In addition, the relative mRNA level of hepatic Ym1 decreased in BDL + DT mice compared with BDL mice (Fig. S7B). The western blotting analysis confirmed that BDL induced upregulation of protein levels of CD11b and iNos was also reduced by DT administration (Fig. 2C and Fig. S7A). Furthermore, DT administration significantly reduced BDL-induced liver injury as indicated by the reduction of serum alanine aminotransferase, aspartate aminotransferase, γ-glutamyltransferase, alkaline phosphatase, total bilirubin, and direct bilirubin (Fig. 2D).

### Macrophage depletion suppresses liver fibrosis and cholangiocyte proliferation in cholestatic livers

**Fig. 3:**
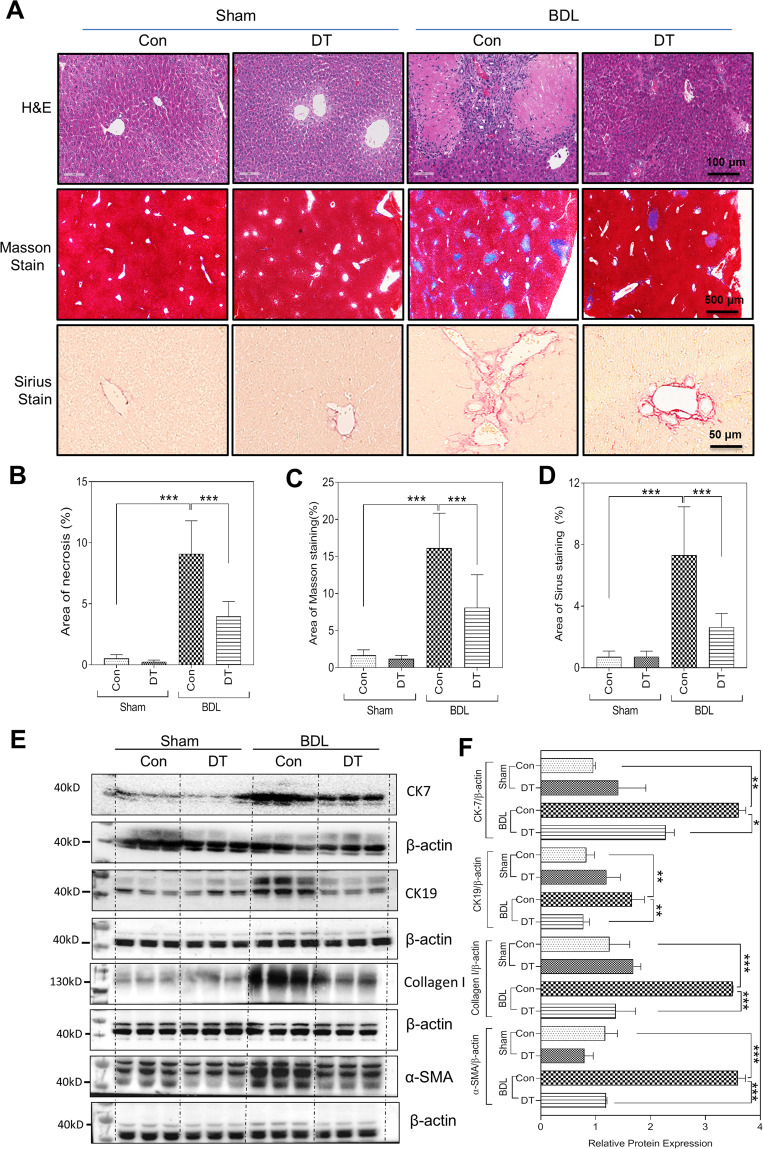
Macrophage depletion inhibits BDL-induced liver fibrosis and cholangiocyte proliferation. **A** Representative images of H&E staining, scale bars: 100 μm; Masson’s trichrome staining, scale bars: 500 μm; and Sirius Red staining scale bars: 50 μm for livers of Sham, Sham + DT, BDL, and BDL + DT mice (*n* = 5–8). **B**–**D** Quantification of necrosis area, Masson area, and Sirius area in panel **A** by ImageJ. **E** Western blotting analysis for CK7, CK19, collagen type I, and α-SMA in Sham, Sham + DT, BDL, and BDL + DT mice. Representative images of the immune blottings are shown. **F** Quantification of panel **E**. Data were expressed as mean ± SD from five to eight mice per group. *P < 0.05, **P < 0.01, ***P < 0.001.

Histologically, hematoxylin–eosin (H&E) staining revealed that the liver necrosis was decreased in BDL + DT mice when compared to that in the BDL mice (Fig. 3A, B). Masson’s trichrome and Sirius red staining indicated that BDL significantly induced liver fibrosis in vehicle-treated mice but had much less impact on DT mice (Fig. 3A, C, D). Moreover, the Ccr2^+^ macrophages in the fibrotic niche decreased in BDL + DT mice compared to those in the BDL mice (Fig. S9A, B). Consistent with these results, BDL-induced increase of the mRNA levels of fibrotic markers, including Col1a1, Acta2, Pdgfb, and Itgb1, was reduced by DT treatment (Fig. S9C). The western blotting analysis further showed that BDL-induced upregulation of protein levels of collagen I and α-SMA was also reduced by DT treatment (Fig. 3E, F). BDL-induced increase of hepatic hydroxyproline levels was also significantly decreased by DT treatment (Fig. S9D). BDL-induced increase of the cytokeratin 19 (CK19)-positive cholangiocytes and accumulation of F4/80^+^ macrophages around bile ducts were also reduced by DT treatment (Fig. S10A, B). The hepatic mRNA levels of cholangiocyte markers, including keratin 7 (Krt7) and Krt19, were significantly increased in BDL mice but reduced by DT treatment (Fig. S10C). The expression of inflammatory genes, including Ccl20, Cxcl3, Tnfa, Il6, and Cxcl12, increased significantly in livers of BDL mice compared to sham mice, but not in livers of BDL + DT mice (Fig. S10C). In addition, the relative mRNA level of hepatic Mki67 decreased in BDL + DT mice compared with BDL mice (Fig. S10C). The western blotting analysis showed that protein levels of both CK7 and CK19 increased markedly in BDL mice livers, but not in BDL + DT mice (Fig. 3E, F). In addition, mRNA levels of angiogenesis marker genes, including epithelial cell adhesion molecule (Epcam), lymphatic vessel endothelial hyaluronan receptor 1 (Lyve1), vascular endothelial growth factor A (Vegfa), angiogenin-1 (Ang1), ICAM1, PECAM, and Clec4g, were reduced in BDL + DT mice when compared to the BDL mice (Fig. S11A). IF staining showed that the hepatic CD31 protein level was increased in BDL mice compared to sham ones, but not in the BDL + DT mice (Fig. S11B, C).

### LncRNA-H19 is involved in macrophage-mediated cholangiocyte proliferation and cholestatic liver fibrosis

**Fig. 4:**
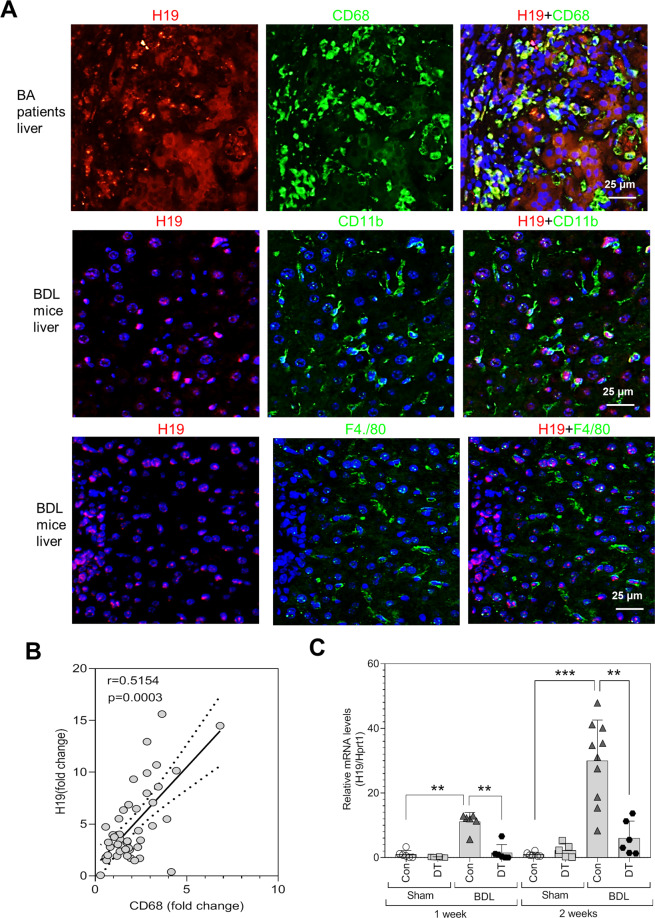
Macrophage depletion reduces the H19 expression in the cholestatic livers. **A** H19 Fluorescence in situ hybridization (FISH) and immunofluorescence (IF) co-staining showed H19 presented in CD68^+^ macrophages in livers of BA patients and H19 FISH and CD11b, F4/80 co-staining in the liver sections of BDL mice. Scale bars: 25 μm. **B** Hepatic levels of CD68 were positively correlated with the expression of H19 in livers of BA patients. **C** Relative mRNA levels of H19 in livers of Sham, Sham + DT, BDL, and BDL + DT (*n* = 5–11) were determined by RT-PCR. Hprt1 was used as an internal control. Data were expressed as mean ± SD. **P < 0.01, ***P < 0.001.

**Fig. 5:**
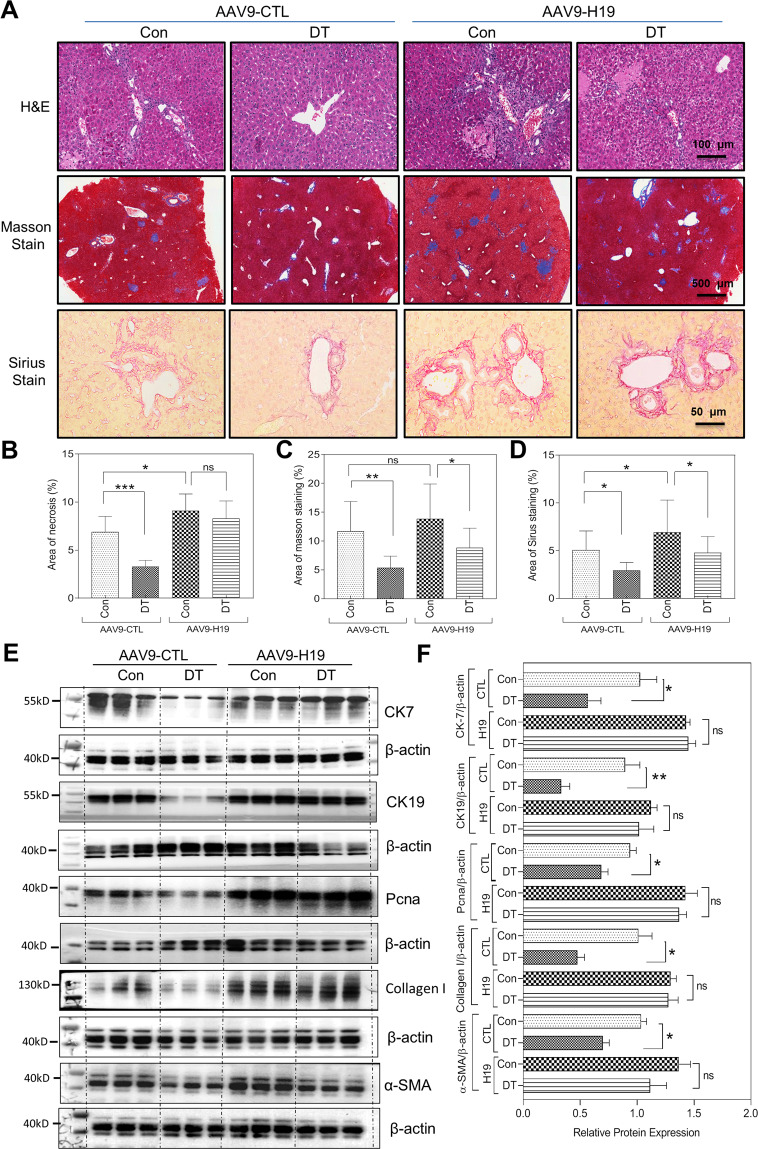
H19 overexpression in the liver counteracts the macrophage depletion-mediated protective effect on cholestatic liver fibrosis and bile duct proliferation. **A** A dose of 1.0 × 10^11^ vector genomes of the AAV9-H19 or controls (AAV9-CTL) were injected into CD11b-DTR mice via tail vein. After 1 week of injection, the mice were subjected to the BDL operation and randomly divided into four groups: AAV9-CTL, AAV9-H19, AAV9-CTL + DT, and AV9-H19 + DT (each group, *n* = 4–6). Representative images of H&E staining, scale bars: 100 μm; Masson’s trichrome staining, scale bars: 500 μm; and Sirius Red staining scale bars: 50 μm for the livers sections from AAV9-CTL, AAV9-H19, AAV9-CTL + DT, and AV9-H19 + DT mice. **B**–**D** Quantification of necrosis area, Masson area, Sirius area in panel **A.** **E** Western blotting analysis for CK7, CK19, Pcna, Collagen I, α-SMA, and β-actin in livers of AAV9-CTL, AAV9-H19, AAV9-CTL + DT, and AAV9-H19 + DT mice. Representative images of the immune blottings are shown. **F** Quantification of panel **E**. Data were expressed as mean ± SD from four to six mice per group. *P < 0.05; **P < 0.01, ***P < 0.001; ns, not significant.

Our previous study reported that long noncoding RNA-H19 (lncRNA-H19) increased in BA livers and played an important role in promoting cholestatic liver fibrosis [17]. Here, we also showed that H19 deficiency reduced the BDL-induced hyperplasia of cholangiocytes and ameliorated liver fibrosis, as indicated by downregulation of the protein expression of CK19, PCNA, α-SMA, and collagen I (Fig. S12A). The fluorescent in situ hybridization assay showed that the H19 was expressed in CD68^+^ macrophages in the livers of BA patients (Fig. 4A). In BDL mice, H19 was detected in both CD11b and F4/80 macrophages (Fig. 4A). The relative mRNA level of hepatic CD68 was correlated with H19 in the livers of BA patients (Fig. 4B). Similar to the previous finding, BDL significantly induced hepatic H19 level, which was reduced markedly in the livers of BDL + DT mice (Fig. 4C). To further determine whether the effect of DT-mediated depletion of macrophages on reducing liver fibrosis is attributed to inhibition of H19, adeno-associated virus serotype 9 (AAV9) overexpressing H19 (AAV9-H19) was used to overexpress H19 in CD11b-DTR mice via tail vein injection. Mice were subjected to BDL 7 days after injection and treated with DT or saline every 2 days for 10 days. The RT-PCR analysis showed that hepatic H19 levels increased about 200-fold in AAV9-H19-treated mice compared to the AAV9-CTL-treated mice (Fig. S13). As shown in Fig. 5, the H&E, Masson’s Trichrome, and Sirius Red staining showed the BDL-induced hepatic necrosis and liver fibrosis were inhibited significantly by giving DT. However, these effects were counteracted or reversed by AAV9-H19 injection (Fig. 5A–D). Consistent with these findings, the western blot analysis indicated that DT injections significantly reduced hepatic protein levels of CK7, CK19, α-SMA, collagen I, and PCNA in BDL mice, which was completely reversed by overexpression of H19 (Fig. 5E, F).

### Macrophage-specific deletion of H19 ameliorates the cholestatic liver injury and fibrosis

**Fig. 6:**
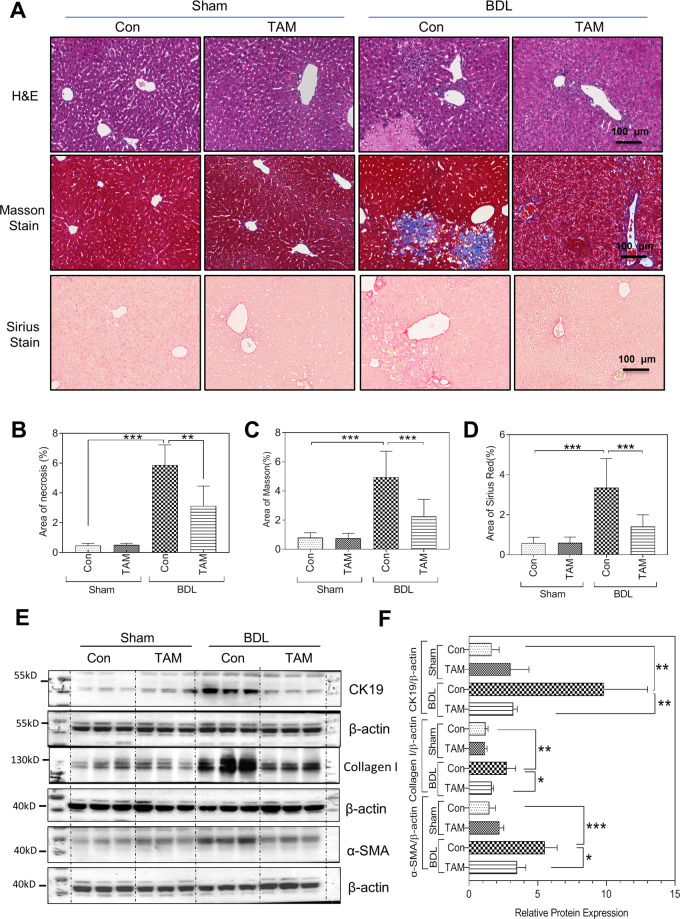
Macrophage-specific depletion of H19 attenuates BDL-induced liver fibrosis. **A** Representative images of H&E staining, Masson’s trichrome staining, Sirius Red staining in the liver sections from Sham, Sham + Tamoxifen, BDL, and BDL + Tamoxifen (TAM) mice (each group, *n* = 3–6). Scale bars: 100 μm. **B**–**D** Quantification of necrosis area, Masson area and Sirius area in the panel **A**. **E** Western blotting analysis for CK19, collagen type I, and α-SMA in Sham, Sham + TAM, BDL, and BDL + TAM mice. Representative images of the immune blotting are shown. **F** Quantification of panel **E**. Data were expressed as mean ± SD from three to six mice per group. *P < 0.05, **P < 0.01, ***P < 0.001.

To further examine the role of macrophage H19 in cholestatic liver injury, H19^fl/fl^: CD11B^CreERT2^ mice were treated with tamoxifen (TAM). As shown in Figs. S2 and  S14A, H19 was successfully deleted in CD11b^+^ cells. The H&E, Masson’s Trichrome, and Sirius Red staining showed that BDL-induced hepatic necrosis and liver fibrosis were were reduced by giving the TAM (Fig. 6A–D). Consistent with these findings, the western blot analysis revealed that TAM injections significantly reduced the protein levels of CK19, α-SMA, and Collagen I in livers of BDL mice (Fig. 6E, F).

### LncRNA-H19 promotes the macrophage activation and polarization in cholestatic livers

**Fig. 7:**
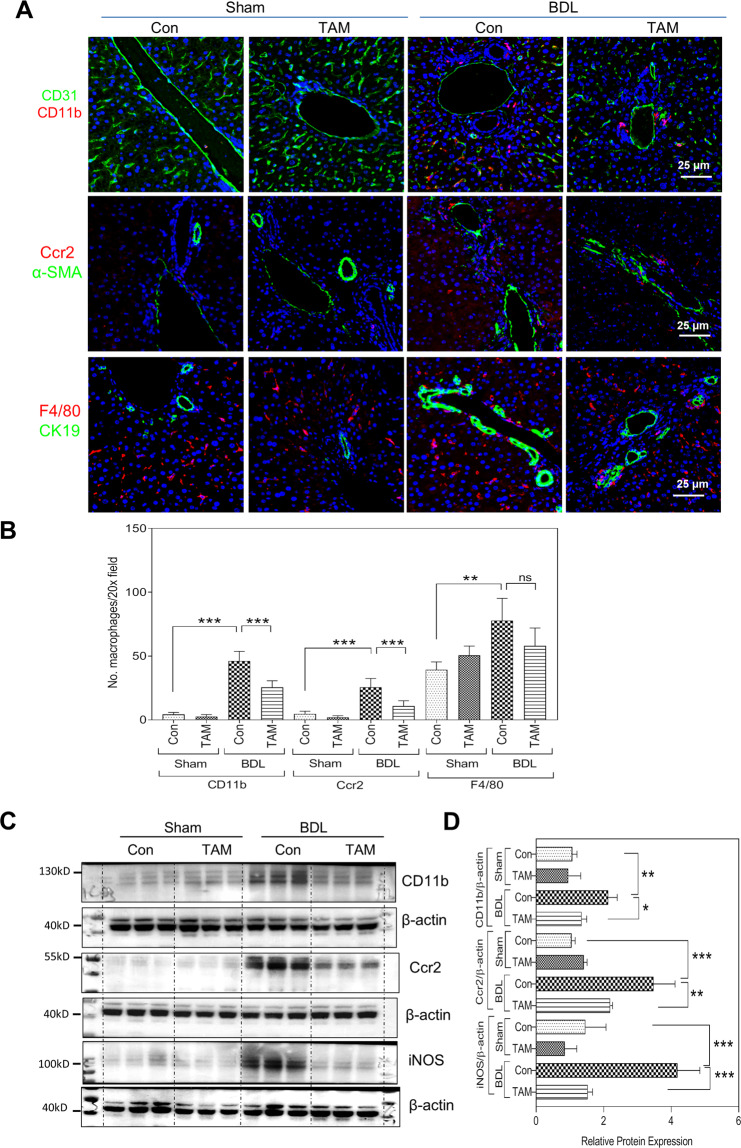
H19 contributes to the activation and polarization of macrophages in the cholestatic livers. **A** Representative images of co-staining of CD31 and CD11b, α-SMA and Ccr2, F4/80 and CK19 in the liver sections from Sham, Sham + TAM, BDL, and BDL + TAM mice (each group, *n* = 3–6). Scale bars: 25 μm. **B** Quantification of CD11b^+^, Ccr2^+^, and F4/80^+^ cells of panel **A**. **C** Western blotting analysis for CD11b, iNOS, Ccr2, and β-actin in the liver tissues from Sham, Sham + TAM, BDL, and BDL + TAM mice. **D** Quantification of panel **B**. Data were expressed as mean ± SD. *P < 0.05; **P < 0.01, ***P < 0.001.

To determine the effects of H19 on the activation and polarization of macrophages in cholestatic livers, we first analyzed the number and polarization of hepatic macrophages in H19 knockout (H19^−^^/−^) and WT mice. We showed that the numbers of CD11b^+^ monocytes/macrophages, F4/80+ macrophages, and Ccr2^+^ macrophages increased significantly in the livers of 2-week-BDL WT mice, but not in that of H19^−/−^ BDL mice (Fig. S15A, B). The western blotting assay indicated that protein levels of CD11b, Ccr2, and iNOS increased significantly in livers of BDL WT mice, but not in the livers of H19^−/−^ BDL mice (Fig. S16A, B). As shown in Fig. 7, IF staining showed the TAM-induced depletion of H19 in macrophages significantly decreased the numbers of CD11b^+^ monocytes/macrophages, F4/80+ macrophages, and Ccr2^+^ macrophages in the livers of BDL mice (Fig. 7A, B). The western blotting analysis confirmed that TAM treatment reduced levels of CD11b, Ccr2, and iNOS proteins in the livers of BDL mice (Fig. 7C, D).

**Fig. 8:**
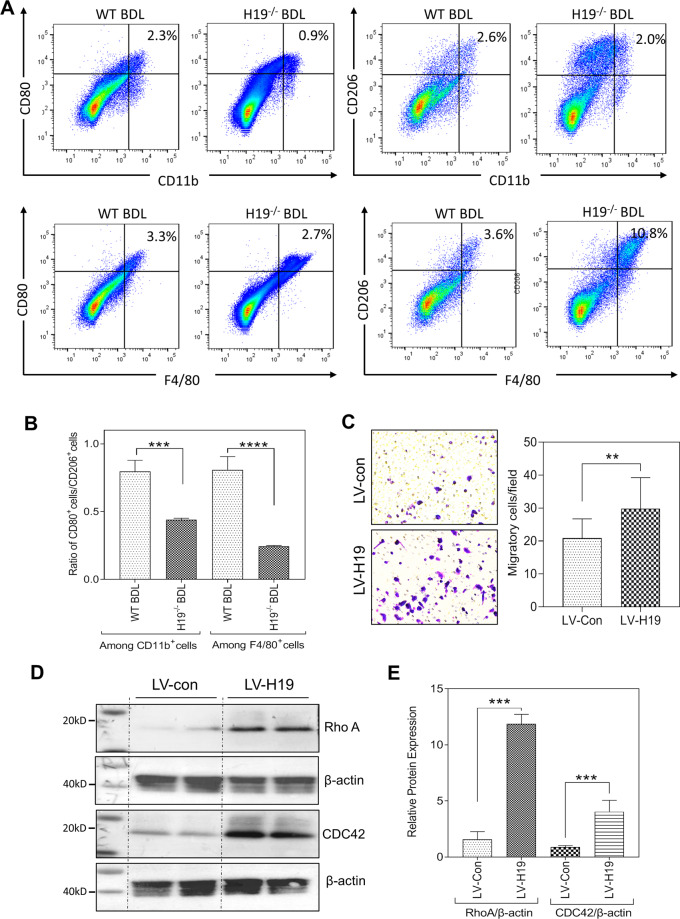
H19 knockout leads to skew macrophage polarization toward M2 subtype. **A** Representative flow cytometry results and images of the percentage of indicated cells in all monocytes are shown. **B** The ratio of CD80/CD206-positive cells in Kupffer cells and monocyte-derived macrophages. **C** Representative images of migration assay are shown. **D** The expression of RhoA and CDC42 in THP-1 macrophages that infected with lentivirus-control (LV-Con) or lentivirus-H19 (LV-H19). **E** Quantification of panel **D**. Results from at least three independent experiments are presented as mean ± SD. *P < 0.05; **P < 0.01, ***P < 0.001.

To further identify the role of H19 in macrophage recruitment and polarization, we used flow cytometry to separate resident Kupffer cells and monocytes, as well as to identify the CD80 and CD206 positive cell populations. As shown in Fig. S17A, B, CD11b^+^ macrophages were decreased significantly in BDL-H19^−/−^ mice when compared with BDL WT mice, but the number of F4/80^+^ macrophages showed no significant change. In contrast to the WT BDL mice, the expression of CD80 was found to be low in F4/80^+^ macrophages and CD11b^+^ macrophages from BDL-H19^−/−^ mice. In F4/80-positive cell population, CD206 increased in BDL-H19^−^^/−^ mice and no significant difference in CD11b-positive CD11b^+^ macrophages population was observed (Fig. 8A). In addition, the ratio of CD80/CD206 was decreased in both F4/80-positive cell population and CD11b-positive cell population (Fig. 8B). We further used the transwell assay to examine the effect of H19 overexpression on cell migration. We overexpressed H19 in THP-1 derived macrophages using H19 recombinant lentivirus. As shown in Fig. 8C, overexpression of H19 promoted the migration of THP-1 derived macrophages. We examined its effect on the expression of the Rho family of small GTPases, including RhoA and cdc42, which play critical roles in cell migration. As shown in Fig. 8D, E, western blot showed H19 overexpression increased protein levels of both RhoA and cdc42).

**Fig. 9:**
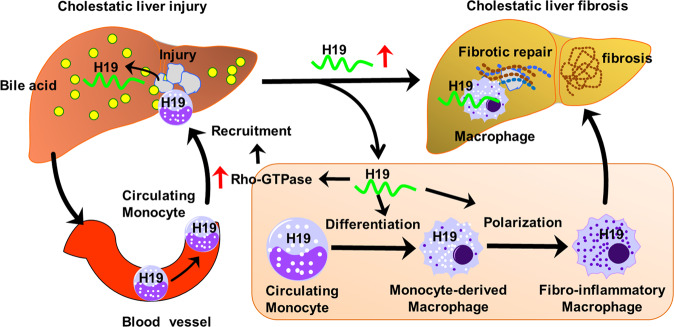
Schematic diagram of mechanisms for macrophage functions in the cholestatic liver fibrosis. The accumulated bile acids caused liver injuries in cholestatic livers, which attracted the monocytes/macrophages from the peripheral circulation to repair damages. Once monocytes/macrophages cells reached the injured areas, H19 increased their activation and polarization and subsequently promoted the liver fibrosis progression. H19 can induce macrophage migration into the injured liver. H19 also promotes macrophage differentiation and polarization in the cholestatic livers.

## Discussion

The development of cholestatic liver fibrosis in BA patients is rapid and severe. However, the limited understanding of cholestatic liver fibrosis mechanisms impedes the development of novel therapies for BA. We here showed that macrophage depletion halts liver fibrosis progression via reducing lncRNA-H19 under cholestatic conditions. We also identified a novel lncRNA-H19 regulatory mechanism in cholestasis-induced liver fibrosis in which lncRNA-H19 enhances activation and polarization of macrophages may occur via the Rho-GTPase pathway. Our study suggests that macrophages may be potential targets in combatting cholestatic liver fibrosis (Fig. 9).

BA is a severe cholestatic liver disease in neonates without effective therapy [3, 4]. Currently, preventing progressive liver injury and fibrosis is essential to their clinical management. The previous studies indicate that macrophage infiltration increasing in BA patients is a key driving force to promote disease progression [5]. The current study showed a significant positive correlation between levels of hepatic CD11B and liver fibrosis, inflammation, and angiogenesis in BA patients. The accumulation of the CD11B^+^ cells and CD68^+^ cells was mainly detected in the fibrotic niche and peribiliary areas in BA patients. In the liver, resident KCs and macrophages recruited from the circulating bone marrow-derived monocyte lineage are two major types of macrophages [20]. In the cholestatic liver, an increase in CCL2 secretion recruits CCR2-expressing cells (such as pro-inflammatory monocytes) to the injury site [8, 21, 22]. It has been reported that Ccr2 knockout mice are protected from cholestatic liver injury [8, 9]. However, the roles of macrophages in liver fibrosis remain unclear. A transgenic mouse (CD11b-DTR) was used in this study to determine the contribution of macrophages to cholestatic liver fibrosis. The previous studies reported that DT injection could selectively deplete most tissue macrophages [12, 13, 18, 19]. We also showed that hepatic macrophages were successfully depleted in CD11b-DTR mice after DT injection in the current study. Macrophage depletion significantly reduced BDL-induced fibrotic liver injury, which was accompanied by inhibition of BDL-induced lncRNA-H19 upregulation. LncRNA-H19 is an imprinted and maternally expressed transcript [23, 24]. Aberrant expression of lncRNA-H19 has been associated with many disease conditions, including various liver diseases [25–29]. It has been reported that H19 is mainly expressed in cholangiocytes under cholestatic conditions, but it can be transported to other hepatic cells via exosomes [15, 16, 30]. Our previous study indicated that H19 expresses in hepatic macrophages and plays a vital role in regulating bile duct cell proliferation and liver fibrosis in BA patients [17]. Similarly, in this study, we identified that H19 was upregulated in CD11b^+^ monocytes/macrophages and F4/80^+^ macrophages in the livers of BDL mice. These results suggest that macrophage H19 is a critical player in promoting cholestatic liver injury. We further confirmed our findings using AAV9-H19 to overexpress H19 in CD11b-DTR mice. As expected, overexpression of H19 counteracted the protective effect of macrophage depletion against BDL-induced liver injury. Conditional deletion of H19 in macrophages using TAM in H19^fl/fl^: CD11B^CreERT2^ mice also ameliorated BDL-induced liver injuries and fibrosis, which is consistent with the findings in the H19 knockout mice. In the absence of H19, the numbers of CD11b^+^ monocytes/macrophages, F4/80+ macrophages, and Ccr2^+^ macrophages are significantly decreased in the livers of BDL mice. H19 deficiency also suppressed the macrophage polarization as indicated by the reduction of the M1 pro-inflammatory mediator, iNOS protein. Similar to the finding in the global H19 knockout mice, macrophage-specific deletion of H19 also decreased the hepatic macrophage infiltration and M1 polarization. A recent study also showed that cholangiocyte-derived H19-exosomes were involved in macrophage activation and hepatic inflammation in cholestatic liver injury [30]. Rho-family GTPases are the key regulators of cell migration and morphogenesis, which are activated by guanine nucleotide exchange factors (GEFs) and inactivated by GTPase-activating proteins (GAPs) [31–33]. It has been reported that Rho-kinase inhibitor Y27632 can reduce BDL-induced cholestatic liver injury [34]. We also identified that H19 overexpression increased the expression of both CDC42 and RhoA in macrophages. However, how H19 regulates the expression of CDC42 and RhoA remains unclear. In hepatocellular carcinoma, it has been reported that H19 positively regulates CDC42 via sponging miR-15b [35]. H19 silencing has suppressed proliferation and invasiveness of hepatocellular cells through miR-15b/CDC42 pathway [35]. It thus hypothesis that H19 regulates the CDC42 and RhoA may through sponging miR-15b in macrophages.

In summary, we demonstrated that hepatic macrophage recruitment and activation are major contributors to cholestatic liver injury both in BA patients and the BDL mouse model. Selective depletion of macrophages attenuates liver fibrosis progression and bile duct proliferation. LncRNA-H19 plays critical roles not only in the cholestasis-induced activation and polarization of macrophages, but also in macrophage recruitment *via* modulating Rho-family GTPases. Targeting H19 represents a potential therapeutic target for cholestatic liver diseases.

## Supplementary information

CDDIS-21-1265R-revised Supplemental with clean version
