# Supplementary material for: Conditional depletion of macrophages ameliorates cholestatic liver injury and fibrosis via lncRNA-H19
Source: Cell Death Dis. 2021 Jun 24;12(7):646. doi: 10.1038/s41419-021-03931-1 (PMC8225916; doi:10.1038/s41419-021-03931-1)

**Supplementary Materials and Methods**

**Materials**

NuPAGE 10% Bis-Tris gel (Cat.No.NP0316BOX, Invitrogen, Carlsbad, CA); SurePAGE 4-20% Bis-Tris gel (Cat.No.M00657, GenScript, Nanjing, China); Hydroxyproline Assay Kit (Cat.No.MAK008-1KT, Sigma-Aldrich, St. Louis, MO); PowerUp SYBR-Green Master Mix kit (Cat. No. A25742) and High Capacity cDNA Reverse Transcription kit (Cat. No. 4368814) were from Applied Biosystems (Foster City, CA); Antibody information is listed in Table S2.

**Histology and immunofluorescence (IF)**

All the liver tissues were immediately fixed in 10% neutral buffered formalin for 24 h and went through dehydration, clearing and paraffin embedding. Sections were cut at 4 µm thick, mounted on positively charged slides, baked at 65 °C for 1h and then stored at room temperature (RT) for later use. Fibrosis was performed using Mason’s Trichrome Stain according to the manufacturer’s protocol (Genmed Scientifics, Wilmington, DE, USA). The collagen fibers were stained blue, the nuclei black and the background red. Fibrosis was also determined by Sirius red stain following the protocol (Servicebio, Wuhan, China). Liver necrosis in liver sections (4-5 μm thick) was assessed by H&E Staining (20 different fields were analyzed from 4 different samples from 4 different animals). We assess liver fibrosis in liver sections (4-5 μm thick) by Sirius red staining and Trichrome staining (20 different fields were analyzed from 4 different samples from 4 different animals). Immunohistochemistry was performed using the chromogen diaminobenzidine as we described previously ^1, 2, 3^. Briefly, the slides were incubated with xylol and descending concentrations of ethanol. Endogenous peroxidases were blocked by using 0.3% H_2_O_2_ for 10 min at RT. After antigen retrieval, blocking was performed using 5% bovine serum albumin for 30 min at RT. The antibodies of αSMA (Servicebio, dilution, 1:500), CD68 (Servicebio, dilution, 1:100), CD11b (Servicebio, dilution, 1:200), CD206 (Servicebio, dilution, 1:500), iNOS (Servicebio, dilution, 1:300), CCR2 (Servicebio, dilution, 1:400), CD31 (Servicebio, dilution, 1:100), CK19 (Servicebio, dilution, 1:150), F4/80 (Servicebio, dilution, 1:300) and Collagen Ⅰ(Abcam, dilution, 1:100) were applied at their optimal concentration overnight in a wet chamber at 4 °C. The slides were rinsed in phosphate-buffered saline (PBS) and incubated with the appropriate secondary antibody for 1 h at RT. Antibody binding was visualized using a liquid diaminobenzidine Substrate Chromogen System (Dako, Glostrup, Denmark). The slides were rinsed in PBS and counterstained with hematoxylin. Immunohistochemistry image analysis was used and the software was Image Pro Plus (Media Cybernetics, Rockville, MD, USA), 10 fields/sample. The number of CD68‐positive cells, CD11B‐positive cells, CCR2‐positive cells, CD206‐positive cells, or iNOS‐positive cells in 2 different samples from 2 different people was counted. The number of CK19‐positive cells, CCR2‐positive cells, CD11B‐positive cells or F4/80‐positive cells in 2 different samples from 2 different animals was counted.

**Transmission electron microscopy (TEM) analysis**

The TEM was analyzed in the Core Facility of Basic Medical Sciences, Shanghai Jiao Tong University School of Medicine. Briefly, the isolated exosomes were concentrated and fixed in 2% paraformaldehyde (PFA) in PBS at room temperature for 10 min. The fixed exosomes were put on the Formvar-carbon-coated grids and air-dried for 20 min. After washing with PBS, the grids were immersed in 1 % glutaraldehyde for 5 min. The exosomes were further embedded in the solution of 4 % uranyl acetate and 2 % methylcellulose (9:1) on ice. The grids were air-dried and analyzed using a Philips CM120 electron microscope at 80 KV by an experienced electron microscopist.

**H19 Fluorescence *in situ* hybridization (FISH) and immunofluorescence (IF) co-staining**

These experiments for human livers were performed according to protocols described in our previous study ^1^. All procedures of the FISH were performed in diethyl pyrocarbonate (DEPC)-treated water. The positive staining signals were observed under the fluorescent microscope (Nikon, Eclipse Ti, Tokyo, Japan). For mice, H19 FISH was performed using a commercially available RNAscope Multiplex Fluorescent Reagent Kit v2 (Advanced Cell Diagnostics, Newark, CA) by following the manufacturer’s instructions. Immunofluorescence staining targeting CD68, CD11b or F4/80 was performed after FISH staining. The positive staining signals were observed using fluorescent microscopy (Nikon, Eclipse Ti, Tokyo, Japan).

**Biochemical measurements**

The mouse blood samples were analyzed for alanine aminotransferase (ALT), aspartate aminotransferase (AST), alkaline phosphatase (ALP), bilirubin, conjugated bilirubin，γ-glutamyl transferase（GGT）and alkaline phosphatase (AKP) by assay kits (Nanjing Jiancheng Bioengineering Institute, Nanjing, China) according to the manufacturer’s instructions.

**Hydroxyproline determination in liver**

A Hydroxyproline Assay Kit was used following the manufacturer’s instructions. Briefly, hydroxyproline content was quantified colorimetrically from 10 mg liver samples. Tissue was homogenized in 100 μL water with 100 μL concentrated hydrochloric acid (HCl, ~12 M) and hydrolyzed at 120°C for 3 hours. Chloramine T/Oxidation Buffer Mixture was added at room temperature for 5 minutes, followed by the addition of Diluted DMAB Reagent and incubation at 60°C for 90 minutes. Measured the absorbance of samples and standards at 560 nm and [hydroxyproline](https://www.sciencedirect.com/topics/medicine-and-dentistry/hydroxyproline) content was expressed as micrograms per gram of liver tissue.

**Quantitative real-time polymerase chain reaction (qRT-PCR)**

Total RNA was extracted from diseased livers of humans and mice using the RNeasy kit (Qiagen, Hilden, Germany) according to the protocol of the manufacturer. RNA amount was determined using Nano-drop spectroscopy (Applied Biosystems, Foster City, CA). High Capacity cDNA Reverse Transcription kit (Applied Biosystems, Foster City, CA) was employed to accomplish reverse transcription. Subsequently, real-time PCR reactions were performed using the ViiA 7 Real-Time PCR System (Applied Biosystems, Foster City, CA) with PowerUp SYBR-Green Master Mix kit (Applied Biosystems, Foster City, CA). PCR reactions were incubated in a 384-well plate at 95°C for 10 min, followed by 40 cycles at 95 °C for 15 s and 60 °C for 1 min. All samples were assayed in triplicate, and data were normalized to endogenous controls HPRT1 and Gapdh. Relative RNA expression levels were calculated using the ^ΔΔ^Ct method. The primers are listed in Table S3.

**Western blotting**

Briefly, about 50 mg tissue was homogenized in 500 μL RIPA buffer (Invitrogen, Carlsbad, CA) supplemented with a protease inhibitor cocktail (Servicebio, Wuhan, China). BCA reagent (Pierce, Rockford, IL, USA) was used to determine the protein concentration. Equal amounts of protein were separated on NuPAGE 10% Bis-Tris gels (Invitrogen, Carlsbad, CA) and transferred onto polyvinylidene diﬂuoride (PVDF) membranes using a dry blotting system (iBLOT system, Invitrogen). After blocking in 5% nonfat milk at room temperature for 60 min, membranes were incubated with the primary antibodies overnight at 4°C.The membranes were washed three times for 30 min with TBST (containing 0.1% Tween-20), and then incubated with secondary antibodies. After ﬁnal washes with TBST, the signals were detected using ECL chemiluminescence reagent kit (Pierce, Rockford, IL, USA). The primary antibodies of alpha smooth muscle actin (1:1000), Cytokeratin 7 (1:500), Cytokeratin 19 (1:500), Collagen I (1:1000), CD11b (1:800), iNOS (1:1000), CCR2 (1:1000), PCNA (1:500), β-actin (1:1500) and β-Tubulin (1:2000) and also Rho-GTPase sample kit (#9968, cell signaling technology) were used in this study.

**Isolation and culture of Kupffer cells**

Kupffer cells were isolated using a two-step collagenase digestion method and cultured as previously described^4^. Briefly, after perfusing of 20 ml Ca^2+^ and Mg^2+^-free Hanks balanced salt solution (G4203-500ML, Servicebio, Wuhan, China) via the portal vein., 25 ml HBSS (G4204-500ML, Servicebio, Wuhan, China) containing 0.05% collagenase Type IV (G5027-100MG, Servicebio, Wuhan, China) was perfused to mice liver at 37°C. After that, the liver was removed and minced with scissors. The liver specimen was filtered through a sterile nylon gauze, parenchymal cells were removed by 3-time centrifugation at 30-50 g for 3 min. Cells were resuspended in buffer, and then gently overlaid on 20:50% percol (40501ES60, Yeasen, Shanghai, China). After centrifuged at 800g for 15 minutes at 4°C, liver MNCs were collected from the interphase and cultured at a density of 1×10^6^ in 24-well culture plates containing RPMI 1640 medium supplemented with 10% FBS and antibiotics (100 U/ml of penicillin G and 100 mg/ml of streptomycin sulphate) at 37°C in the presence of 5% CO_2_. After 1 hour, nonadherent cells were removed by replacing the buffer.

**Flow Cytometry**

After the two-step collagenase digestion, hepatic non-parenchymal cells were collected and Intracellular staining was performed with anti-CD206-APC or anti-CD80-APC antibody, followed by incubation with specific fluorescence antibodies (F4/80, CD11b) for 30 min at 4°C. Data were acquired on a FACSCanto II flow cytometer (BD Biosciences, San Jose, CA, USA). Further analysis was performed using FlowJo software (Tree Star Inc, Ashland, OR, USA). Detailed information for antibodies used in flow cytometry analysis is provided in Supplementary Table S2.

**THP-1 cell culture, macrophage differentiation and H19 overexpression**

THP-1 cell lines were purchased from the American Type Culture Collection (ATCC, Rockville, MD) and were maintained in RPMI 1640 medium containing 10% fetal bovine serum (FBS) and 0.05 mM 2-mercaptoethanol (Sigma-Aldrich, Saint Louis, MO). The human H19 (NR_002196) cDNA was cloned into lentivirus vector and transfected into 293T cells for the virus package (GeneChem, Shanghai, China). The THP-1 cells were infected with lentivirus-containing control (LV-Con) and H19 (LV-H19) for 5 days and the protein was extracted to perform Western-blot. Using 100 ng/ml phorbol 12-myristate 13-acetate (PMA; Sigma-Aldrich, Saint Louis, MO) in 6-well cell culture plates with 3 ml cell suspension in each well to obtain macrophage-like state. After differentiation, adherent cells were washed twice in medium (RPMI 1640 medium without PMA), and then placed for 48 h, leaving the macrophages in a resting state. Cells were also infected with lentivirus-containing control (LV-Con) and H19 (LV-H19) for 5 days for the further experiments.

**Macrophage Migration Assay**

THP-1 cells infected with the H19 recombinant lentivirus or negative control virus and mouse primary Kupffer cells plated (5 × 10^4^ per well) in top chambers of 24-well plates. The top chambers were incubated in medium with 2% FBS while the bottom chambers were full with medium with 10% FBS. After 48h culture, images were captured using Leica microscope model (Tokyo, Japan).

**Supplemental Reference**

1. Xiao Y, Liu R, Li X, Gurley EC, Hylemon PB, Lu Y*, et al.* Long Noncoding RNA H19 Contributes to Cholangiocyte Proliferation and Cholestatic Liver Fibrosis in Biliary Atresia. *Hepatology* 2019, **70**(5)**:** 1658-1673.

2. Liu R, Li X, Huang Z, Zhao D, Ganesh BS, Lai G*, et al.* C/EBP homologous protein-induced loss of intestinal epithelial stemness contributes to bile duct ligation-induced cholestatic liver injury in mice. *Hepatology* 2018, **67**(4)**:** 1441-1457.

3. Liu R, Li X, Zhu W, Wang Y, Zhao D, Wang X*, et al.* Cholangiocyte-Derived Exosomal Long Noncoding RNA H19 Promotes Hepatic Stellate Cell Activation and Cholestatic Liver Fibrosis. *Hepatology* 2019, **70**(4)**:** 1317-1335.

4. Kinoshita M, Uchida T, Sato A, Nakashima M, Nakashima H, Shono S*, et al.* Characterization of two F4/80-positive Kupffer cell subsets by their function and phenotype in mice. *J Hepatol* 2010, **53**(5)**:** 903-910.

**Table S1 The information of patients with biliary atresia and control subjects**

| **NO** | **Gender** | **Age** | **T-Bilirubin (μM)** | **D-Bilirubin (μM)** | **Bile Acid (μM)** | **ALT (U/L)** | **AST (U/L)** | **γGT (U/L)** |
| --- | --- | --- | --- | --- | --- | --- | --- | --- |
|  |  |  |  |  |  |  |  |  |
| BA 1 | M | 2m | 205.9 | 164.1 | 133.4 | 250 | 269 | 315 |
| BA 2 | F | 3m | 218.6 | 118.7 | 62.1 | 266 | 365 | 298 |
| BA 3 | F | 2m | 216.3 | 119.8 | 90.6 | 645 | 1194 | 128 |
| BA 4 | F | 3m | 196.6 | 96.7 | 92.5 | 244 | 456 | 412 |
| BA 5 | M | 3m | 107.6 | 83.8 | 114.4 | 123 | 121 | 154 |
| BA 6 | M | 2m | 193.2 | 108.2 | 68.3 | 412 | 822 | 390 |
| BA 7 | F | 1m | 143.7 | 73.6 | 96.9 | 475 | 728 | 317 |
| BA 8 | M | 2m | 181.8 | 137.6 | 133.1 | 190 | 481 | 1240 |
| BA 9 | M | 1m | 161.5 | 48.4 | 27.7 | 80 | 186 | 744 |
| BA 10 | M | 2m | 126.1 | 69.2 | 65.8 | 113 | 270 | 768 |
| BA 11 | F | 1m | 167.6 | 89 | 49.3 | 119 | 269 | 399 |
| BA 12 | F | 3m | 177.6 | 73.8 | 66.4 | 436 | 441 | 318 |
| BA 13 | M | 3m | 156.3 | 50.3 | 15.5 | 162 | 296 | 380 |
| BA 14 | F | 3m | 109.4 | 34.2 | 164.2 | 190 | 175 | 1371 |
| BA 15 | F | 3m | 136.2 | 71.2 | 73.9 | 218 | 390 | 543 |
| BA 16 | M | 2m | 125.5 | 57.9 | 92.4 | 247 | 523 | 204 |
| BA 17 | M | 17d | 274.6 | 167.4 | 130.9 | 173 | 314 | 422 |
| BA 18 | M | 2m | 115.2 | 81.8 | 94.8 | 94 | 135 | 184 |
| BA 19 | M | 2m | 156.3 | 87.1 | 95 | 440 | 723 | 107 |
| BA 20 | M | 2m | 208.7 | 159.7 | 115.1 | 77 | 119 | 1877 |
| BA 21 | F | 2m | 149.2 | 78.5 | 90.7 | 124 | 291 | 707 |
| BA 22 | M | 2m | 162.7 | 81.2 | 114 | 151 | 167 | 203 |
| BA 23 | F | 2m | 130.8 | 54.7 | 162 | 107 | 138 | 177 |
| BA 24 | F | 2m | 137.5 | 99.5 | 199.4 | 161 | 265 | 261 |
| BA 25 | M | 2m | 267.3 | 165.4 | 91.6 | 79 | 183 | 223 |
| BA 26 | F | 2m | 93.9 | 75.2 | 110.2 | 63 | 118 | 321 |
| BA 27 | M | 2m | 124.5 | 98.2 | 117 | 56 | 78 | 101 |
| BA 28 | M | 2m | 163.6 | 124 | 114.4 | 217 | 306 | 1006 |
| BA 29 | M | 2m | 32.1 | 0 | 5.2 | 12 | 32 | 1225 |
| BA 30 | F | 2m | 151.4 | 54.6 | 107.6 | 239 | 569 | 182 |
| BA 31 | F | 1m | 157.9 | 61.4 | 98.2 | 166 | 155 | 154 |
| BA 32 | M | 17d | 144.3 | 110.2 | 96.3 | 80 | 133 | 289 |
| BA 33 | F | 2m | 144.1 | 117.7 | 99.7 | 140 | 286 | 665 |
| BA 34 | M | 2m | 163.6 | 137.8 | 133.4 | 99 | 158 | 381 |
| BA 35 | F | 2m | 157.1 | 109.1 | 118.8 | 94 | 141 | 461 |
| BA 36 | M | 2m | 252.5 | 225.9 | 125.5 | 99 | 306 | 98 |
| BA 37 | F | 2m | 127.2 | 101.4 | 106.8 | 78 | 153 | 1017 |
| BA 38 | F | 2m | 193.8 | 93.6 | 106 | 344 | 502 | 139 |
| BA 39 | F | 49d | 101.6 | 84.3 | 56.9 | 164 | 242 | 680 |
| BA 40 | F | 1m | 191 | 92.1 | 123.6 | 97 | 214 | 252 |
| BA 41 | M | 2m | 184.7 | 82.6 | 128.6 | 131 | 276 | 1051 |
| BA 42 | F | 2m | 165.6 | 77.2 | 197.1 | 100 | 185 | 417 |
| BA 43 | M | 3m | 145.8 | 116.1 | 141.5 | 188 | 239 | 383 |
| BA 44 | F | 2m | 138.5 | 74.1 | 118.5 | 128 | 128 | 787 |
| HC 1 | M | 3m | 1.5 | 18 | 2.3 | 34 | 38 | 20 |
| HC 2 | M | 3m | 18.5 | 4.6 | 12 | 112 | 111 | 225 |
| HC 3 | F | 2m | 10.5 | 4.7 | 4.5 | 176 | 160 | 352 |
| HC 4 | F | 3m | 9.2 | 11.5 | 3.1 | 126 | 121 | 21 |
| HC 5 | F | 5m | 3 | 12.4 | 5.2 | 85 | 29 | 121 |
| HC 6 | F | 2m | 6.2 | 5.4 | 4.2 | 85 | 29 | 26 |
| HC 7 | M | 6m | 2.4 | 5 | 1.1 | 81 | 34 | 66 |
| HC 8 | M | 3m | 3.1 | 9.3 | 1.3 | 58 | 62 | 64 |
| HC 9 | F | 42d | 77 | 6.7 | 6.6 | 67 | 88 | 127 |
| HC 10 | F | 7m | 6.6 | 5 | 0 | 38 | 54 | 114 |
| HC 11 | F | 4m | 3.5 | 1.9 | 13.4 | 40 | 60 | 57 |
| HC 12 | F | 6m | 13.9 | 1.7 | 0.9 | 53 | 55 | 92 |

BA, bile acid (0-10 μM); ALT, alanine aminotransferase (0-75 U/L);AST, aspartate aminotransferase (8-38 U/L); γGT ,γ-glutamyltranspeptidase (16-73 U/L); T-Bilirubin, total bilirubin (3.42-20.52 μM); D-Bilirubin, direct bilirubin (0-6.8).

**Table S2 Antibody information**

| Antibody | Source | Catalog# | Application/dilution |
| --- | --- | --- | --- |
| beta-tubulin | Bioss Inc | bs-33034M | WB(1:2000) |
| alpha smooth muscle Actin | Abcam | ab32575 | WB(1:1000) |
| alpha smooth muscle Actin | Bioss Inc | bs-0189R | WB(1:1000) |
| Cytokeratin 7 | Bioss Inc | BS-1610R | WB(1:1000) |
| Cytokeratin 7 | Abcam | ab9021 | WB(1:500) |
| Collagen I | Abcam | ab138492 | WB(1:1000) |
| Collagen I | Bioss Inc | bs-0578R | WB(1:1000) |
| CK19 | Servicebio | GB11197 | WB(1:500) |
| CD11b | Bioss Inc | bs-1014R | WB(1:800) |
| iNOS | Bioss Inc | bs-20601R | WB(1:1000) |
| CCR2 | Bioss Inc | bs-10963R | WB(1:1000) |
| PCNA | Cell Signaling Technology | 2586S | WB(1:500) |
| beta-Actin | Cell Signaling Technology | 4970S | WB(1:1500) |
| αSMA | Servicebio | GB13044 | IHC/IF(1:500) |
| CD68 | Servicebio | GB13067-2 | IHC/IF(1:100) |
| CD68 | Proteintech | 66231-2-IG | IHC/IF(1:3000) |
| CD11b | Servicebio | GB11058 | IHC/IF(1:200) |
| CD206 | Servicebio | GB11062 | IHC/IF(1:500) |
| iNOS | Servicebio | GB11119 | IHC/IF(1:300) |
| CCR2 | Servicebio | GB11326 | IHC/IF(1:300) |
| CD31 | Servicebio | GB13063 | IHC/IF(1:100) |
| Collagen I | Servicebio | GB11022-3 | IHC/IF(1:1000) |
| CK19 | Servicebio | GB12197 | IHC/IF(1:1000) |
| F4/80 | Servicebio | GB11027 | IHC/IF(1:300) |
| APC-CD80 | eBioscience | 17-0801-82 | Flow Cytometry(1：50) |
| APC-CD206 | Biolegend | 141707 | Flow Cytometry(1：25) |
| PE-F4/80 | eBioscience | 12-4801-80 | Flow Cytometry(1：50) |
| FITC-CD11b | Biolegend | 101206 | Flow Cytometry(1：50) |

**Table S3 The primer sequences for qRT-PCR**

| **The sequences of human primers** | | |
| --- | --- | --- |
| HPRT1 | Forward (5'-3') | TTGCTTTCCTTGGTCAGGCA |
|  | Reverse (5'-3') | ATCCAACACTTCGTGGGGTC |
| ITGAM | Forward (5'-3') | GCTTTGGTGGCTTCCTTGTG |
|  | Reverse (5'-3') | CTGGAAGGAGCCAGAACCTG |
| CD68 | Forward (5'-3') | GACAGCCTAGCTGGACTTTGG |
|  | Reverse (5'-3') | TGTGAGGACAGTCATTCCCTG |
| NOS2 | Forward (5'-3') | CGCATGACCTTGGTGTTTGG |
|  | Reverse (5'-3') | CATAGACCTTGGGCTTGCCA |
| CCL2 | Forward (5'-3') | GATCTCAGTGCAGAGGCTCG |
|  | Reverse (5'-3') | TTTGCTTGTCCAGGTGGTCC |
| CCR2 | Forward (5'-3') | GGAGAGCAGAGAGTGGAAATGT |
|  | Reverse (5'-3') | GTGTGAGTCAGGCAAACCCT |
| CCL20 | Forward (5'-3') | TTGTCTGTGTGCGCAAATCC |
|  | Reverse (5'-3') | TTGGACAAGTCCAGTGAGGC |
| CXCL3 | Forward (5'-3') | AGCCACACTCAAGAATGGGA |
|  | Reverse (5'-3') | ACCCTGCAGGAAGTGTCAATG |
| IL1B | Forward (5'-3') | CAGAAGTACCTGAGCTCGCC |
|  | Reverse (5'-3') | AGATTCGTAGCTGGATGCCG |
| IL6 | Forward (5'-3') | TGCAATAACCACCCCTGACC |
|  | Reverse (5'-3') | GTGCCCATGCTACATTTGCC |
| CXCL8 | Forward (5'-3') | CACTGCGCCAACACAGAAAT |
|  | Reverse (5'-3') | GCTTGAAGTTTCACTGGCATC |
| IL10 | Forward (5'-3') | TGAAAACAAGAGCAAGGCCG |
|  | Reverse (5'-3') | GCCACCCTGATGTCTCAGTT |
| PECAM | Forward (5'-3') | GAGGGGCCACATGCATCTAT |
|  | Reverse (5'-3') | CCTGCTCGGTTCTCTCTGTG |
| CLEC4G | Forward (5'-3') | CCAGTGAGAACAGGCGTGAA |
|  | Reverse (5'-3') | AGGGCCTGTACCTGGTGG |
| ANPEP | Forward (5'-3') | TGGAGGGGCAGGGACG |
|  | Reverse (5'-3') | GGAGATCCAGGAACGGTGTG |
| ACTA2 | Forward (5'-3') | AAAGCAAGTCCTCCAGCGTT |
|  | Reverse (5'-3') | TAGTCCCGGGGATAGGCAAA |
| COL1A1 | Forward (5'-3') | AGTGGTTTGGATGGTGCCAA |
|  | Reverse (5'-3') | GCACCATCATTTCCACGAGC |
| CFTR | Forward (5'-3') | TGGATCGCTCCTTTGCAAGT |
|  | Reverse (5'-3') | AAGTCCACAGAAGGCAGACG |
| ICAM1 | Forward (5'-3') | TCTTCCTCGGCCTTCCCATA |
|  | Reverse (5'-3') | AGGTACCATGGCCCCAAATG |
| TNFA | Forward (5'-3') | CACAGTGAAGTGCTGGCAAC |
|  | Reverse (5'-3') | AGGAAGGCCTAAGGTCCACT |
| KRT19 | Forward (5'-3') | CACCAGCCGGACTGAAGAAT |
|  | Reverse (5'-3') | GCAGGTCAGTAACCTCGGAC |
| **The sequences of mouse primers** | | |
| GAPDH | Forward (5'-3') | CCCTTAAGAGGGATGCTGCC |
|  | Reverse (5'-3') | TACGGCCAAATCCGTTCACA |
| ITGAM | Forward (5'-3') | AGCTTGGCTTTTTCAAGCGG |
|  | Reverse (5'-3') | AAAGGCCGTTACTGAGGTGG |
| CD68 | Forward (5'-3') | GGGGCTCTTGGGAACTACAC |
|  | Reverse (5'-3') | GTACCGTCACAACCTCCCTG |
| HPRT1 | Forward (5'-3') | CAGTCCCAGCGTCGTGATTA |
|  | Reverse (5'-3') | TGGCCTCCCATCTCCTTCAT |
| CCL20 | Forward (5'-3') | ATGGCCGATGAAGCTTGTGA |
|  | Reverse (5'-3') | CTCCTTGGGCTGTGTCCAAT |
| NOS2 | Forward (5'-3') | TGGTGAAGGGACTGAGCTGT |
|  | Reverse (5'-3') | GCTACTCCGTGGAGTGAACA |
| CD206 | Forward (5'-3') | GTGGAGTGATGGAACCCCAG |
|  | Reverse (5'-3') | CTGTCCGCCCAGTATCCATC |
| KRT19 | Forward (5'-3') | CTCAGACCTGCGTCCCTTTT |
|  | Reverse (5'-3') | CCGTACCCCCAAAGGAAGAC |
| KRT7 | Forward (5'-3') | CCGGAATGAGATTGCGGAGA |
|  | Reverse (5'-3') | GGACTCTAACTTGGCACGCT |
| MKI67 | Forward (5'-3') | GAGCTAACTTGCGCTGACTG |
|  | Reverse (5'-3') | CGGAGAAGCCTCTCGGTGAA |
| COL1A1 | Forward (5'-3') | CGACCTCAAGATGTGCCACT |
|  | Reverse (5'-3') | CCATCGGTCATGCTCTCTCC |
| ACTA2 | Forward (5'-3') | CCGACCGAATGCAGAAGGA |
|  | Reverse (5'-3') | ACAGAGTATTTGCGCTCCGAA |
| H19 | Forward (5'-3') | GGGTCTGTTTCTTTACTT |
|  | Reverse (5'-3') | TAGCACCATTTCTTTCAT |
| F4/80 | Forward (5'-3') | TGTCTGAAGATTCTCAAAACATGGA |
|  | Reverse (5'-3') | TGGAACACCACAAGAAAGTGC |
| CCR2 | Forward (5'-3') | GCCATCATAAAGGAGCCATACC |
|  | Reverse (5'-3') | ATGCCGTGGATGAACTGAGG |
| CXCL3 | Forward (5'-3') | GAAAGGAGGAAGCCCCTCAC |
|  | Reverse (5'-3') | ACACATCCAGACACCGTTGG |
| IL6 | Forward (5'-3') | CAACGATGATGCACTTGCAGA |
|  | Reverse (5'-3') | GTGACTCCAGCTTATCTCTTGGT |
| TNFα | Forward (5'-3') | CGTGCTCCTCACCCACAC |
|  | Reverse (5'-3') | GGGTTCATACCAGGGTTTGA |
| CXCL12 | Forward (5'-3') | GGTGCTCAAACCTGACGGTA |
|  | Reverse (5'-3') | GGCAGCTCCTCTTTGGCTTA |
| YM1 | Forward (5'-3') | GGGCCCTTATTGAGAGGAGC |
|  | Reverse (5'-3') | CCAGCTGGTACAGCAGACAA |
| PDGFB | Forward (5'-3') | CCACCCTCTAGCTTCGTTGC |
|  | Reverse (5'-3') | GGCTCGGGTCAGTCTGTCTA |
| ITGB1 | Forward (5'-3') | CCTGTAACTCCGACGCCTTT |
|  | Reverse (5'-3') | AAGGTCCCCACTCAGCAATG |
| EPCAM | Forward (5'-3') | AACACAAGACGACGTGGACA |
|  | Reverse (5'-3') | GCTCTCCGTTCACTCTCAGG |
| LCAM1 | Forward (5'-3') | CTGGGCTTGGAGACTCAGTG |
|  | Reverse (5'-3') | CCACACTCTCCGGAAACGAA |
| PECAM1 | Forward (5'-3') | GAGCCTCACCAAGAGAACGG |
|  | Reverse (5'-3') | CCCAACATGAACAAGGCAGC |
| LYVE1 | Forward (5'-3') | CCAGAATCCACTGACCCCAC |
|  | Reverse (5'-3') | GGTGCCAAGCATTTCGGTTT |
| VEGFA | Forward (5'-3') | TATTCAGCGGACTCACCAGC |
|  | Reverse (5'-3') | AACCAACCTCCTCAAACCGT |
| ANG1 | Forward (5'-3') | CCTTAGCATAGGGGCACACT |
|  | Reverse (5'-3') | TGTGTAACCGTTCAGCGTGG |
| CLEC4G | Forward (5'-3') | AAGGCTACCGGTGGGTAGAT |
|  | Reverse (5'-3') | GGCGCTAGGCTTAGTAGCAA |

**Supplementary Figures**

**Fig.S1. Structure of the CD11b-DTR transgene and the genotype of CD11b-DTR mice and H19 knockout mice (H19^-/-^) mice.** (A) Diagram shows the gene construct. DTR-eGFP fusion gene cDNA was inserted between the human CD11b promoter (The coordinates are -1,704 to +83 relative to transcription start point) and human growth hormone (hGH) sequence providing splicing and polyadenylation sequences. Right-facing arrow indicates transcription start point. Representative gel images for CD11b-DTR genotype using the PCR analysis. WT, wild type controls DNA. (B) Representative gel images for H19^-/-^ (H19 Δ Exon1-5) genotype using the PCR analysis. WT, wild type control DNA; H_2_O, negative control.


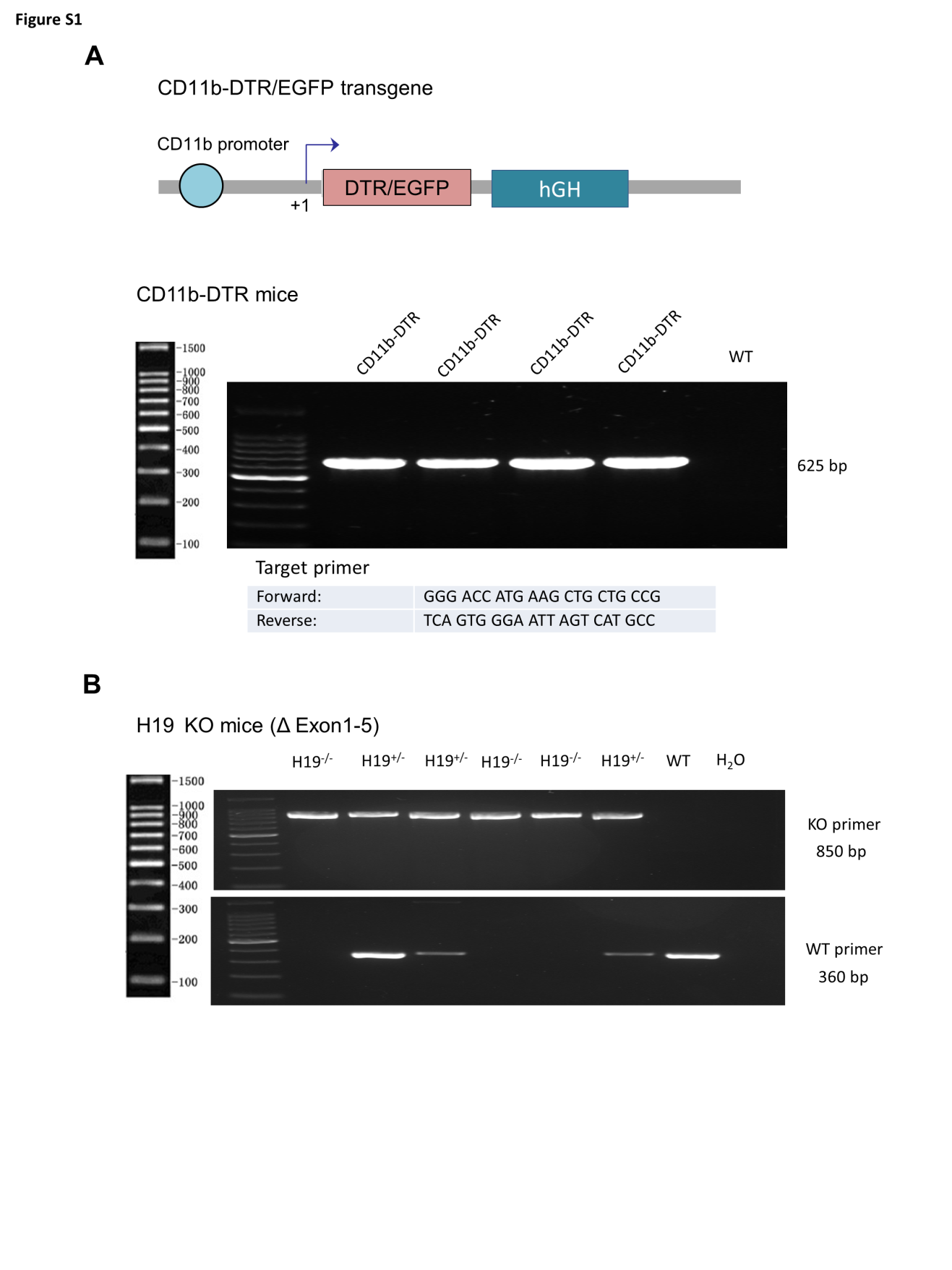


**Fig.S2. Gene structure and genotypes of the H19^flox/flox^ and CD11b^CreERT2^ transgene.** (A) Structure of the H19^flox/flox^ transgene in mice. Representative gel images for H19^flox/flox^ genotype. (B) Structure of the CD11b^CreERT2^ transgene in mice. Representative gel images for CD11b^CreERT2^ genotype. WT, wild type control DNA; H_2_O, negative control.

**
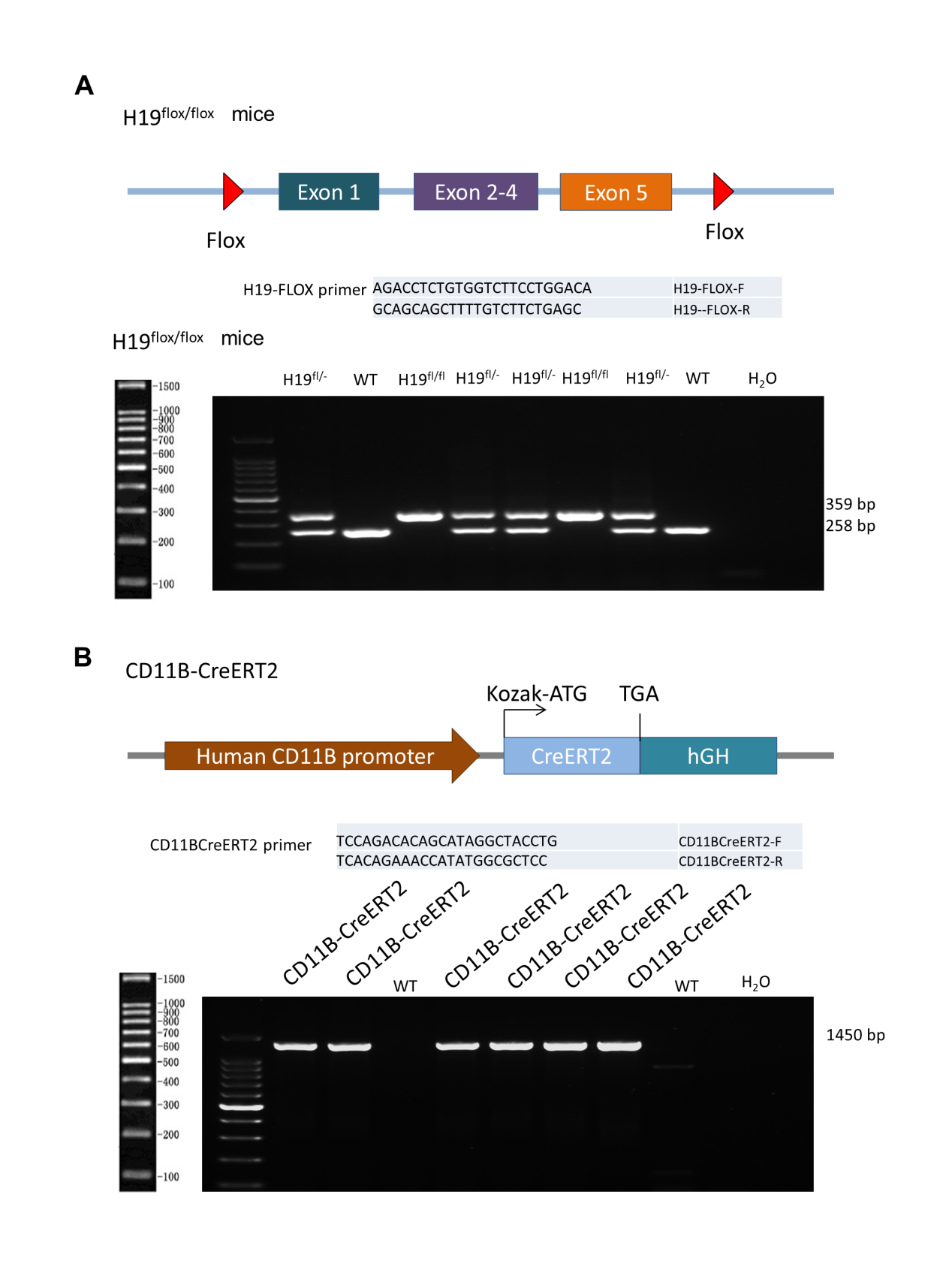
**

**Fig.S3. The mouse H19 cDNA cloned into AAV9 plasmid.** (A) The mouse cDNA (NR_130973) was cloned into AAV9 plasmid via BamHI and HindIII. (B) The sequencing results confirmed the H19 cDNA cloned into AAV9 plasmid successfully. The red parts show the sequencing results of H19 cDNA.

**
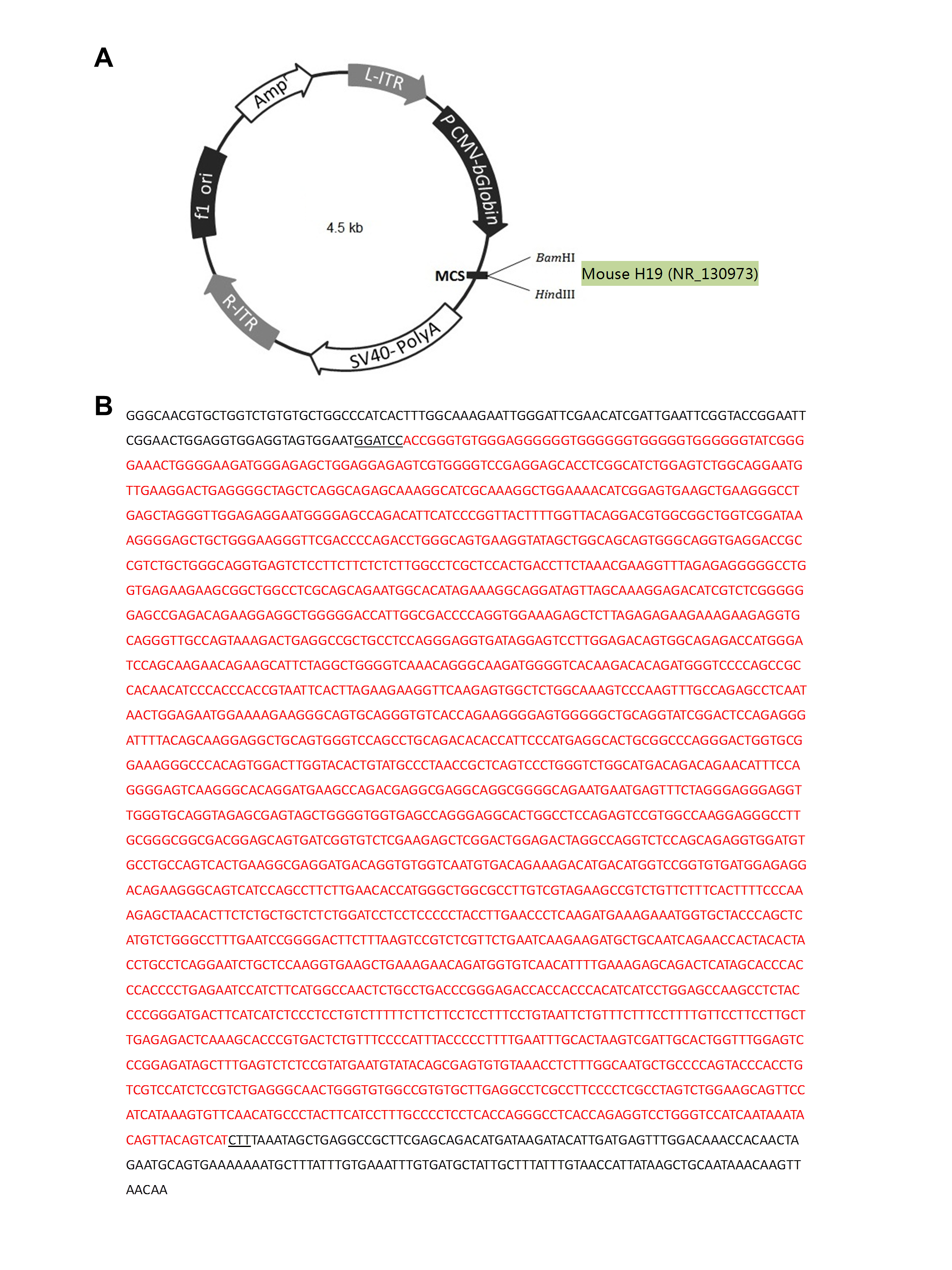
**

**Fig.S4.** **Macrophages accumulated around the fibrotic areas in livers of BA patients.** Representative pictures of TEM analysis showed that macrophages presented in the hepatic vein and fibrotic niche. Red arrows show macrophages. Green arrows show the fibrotic niche. Scale bars: 5 μm and 2 μm.

**
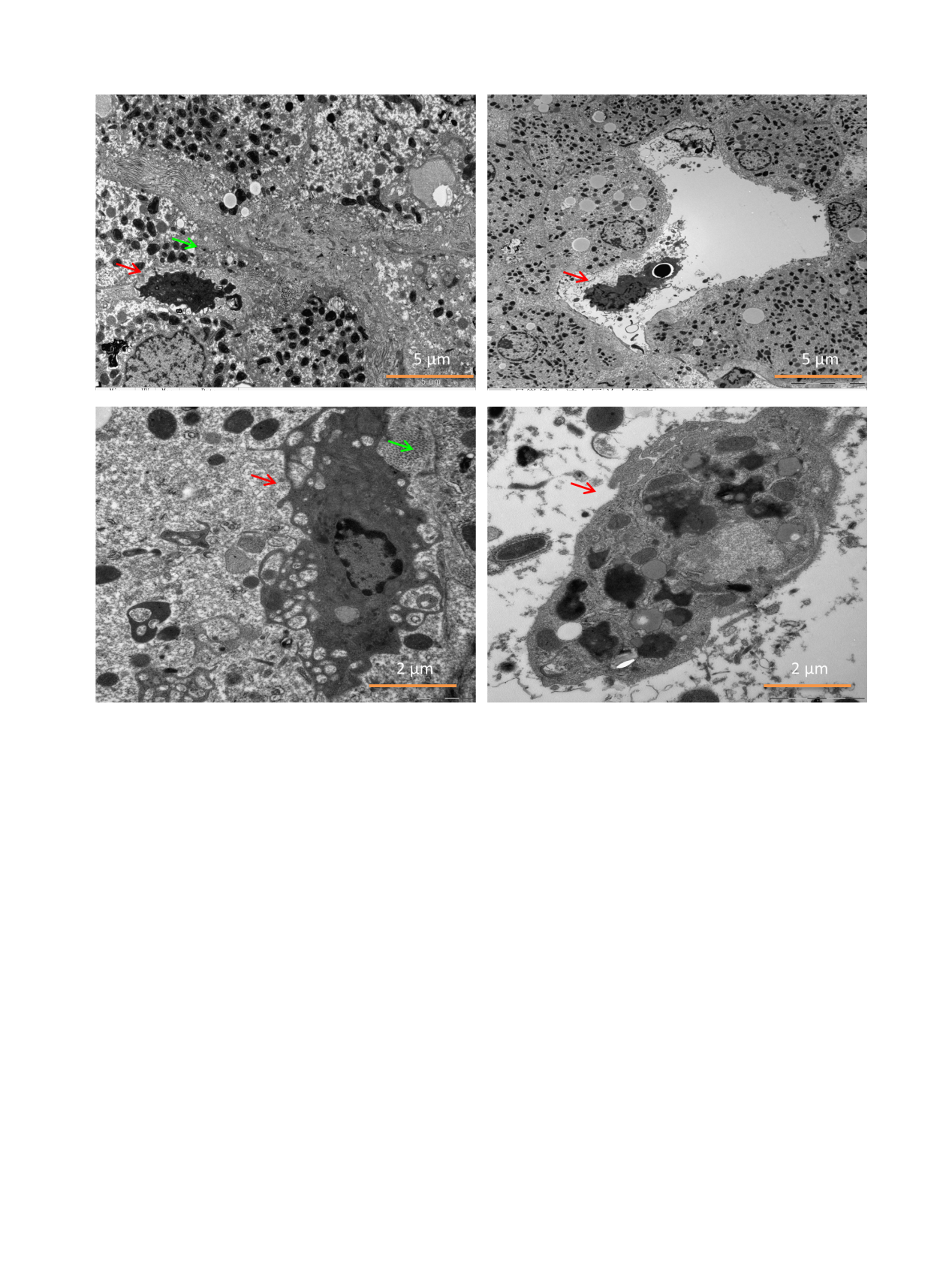
**

**Fig.S5. Increased levels of macrophages’ genes in BA patients.** Relative mRNA levels of markers for macrophages, inflammation, fibrosis, and angiogenesis in livers of BA patients (n = 44) and controls (n = 12) were determined by real-time PCR (RT-PCR). HPRT1 was used as an internal control. Data were expressed as mean ± SD. Statistical significance relative to controls: *P < 0.05; **P<0.01; ***P<0.001; ns, not significant. Abbreviations: BA, biliary atresia; HC, healthy controls; HPRT1, hypoxanthine phosphoribosyl transferase 1.

**
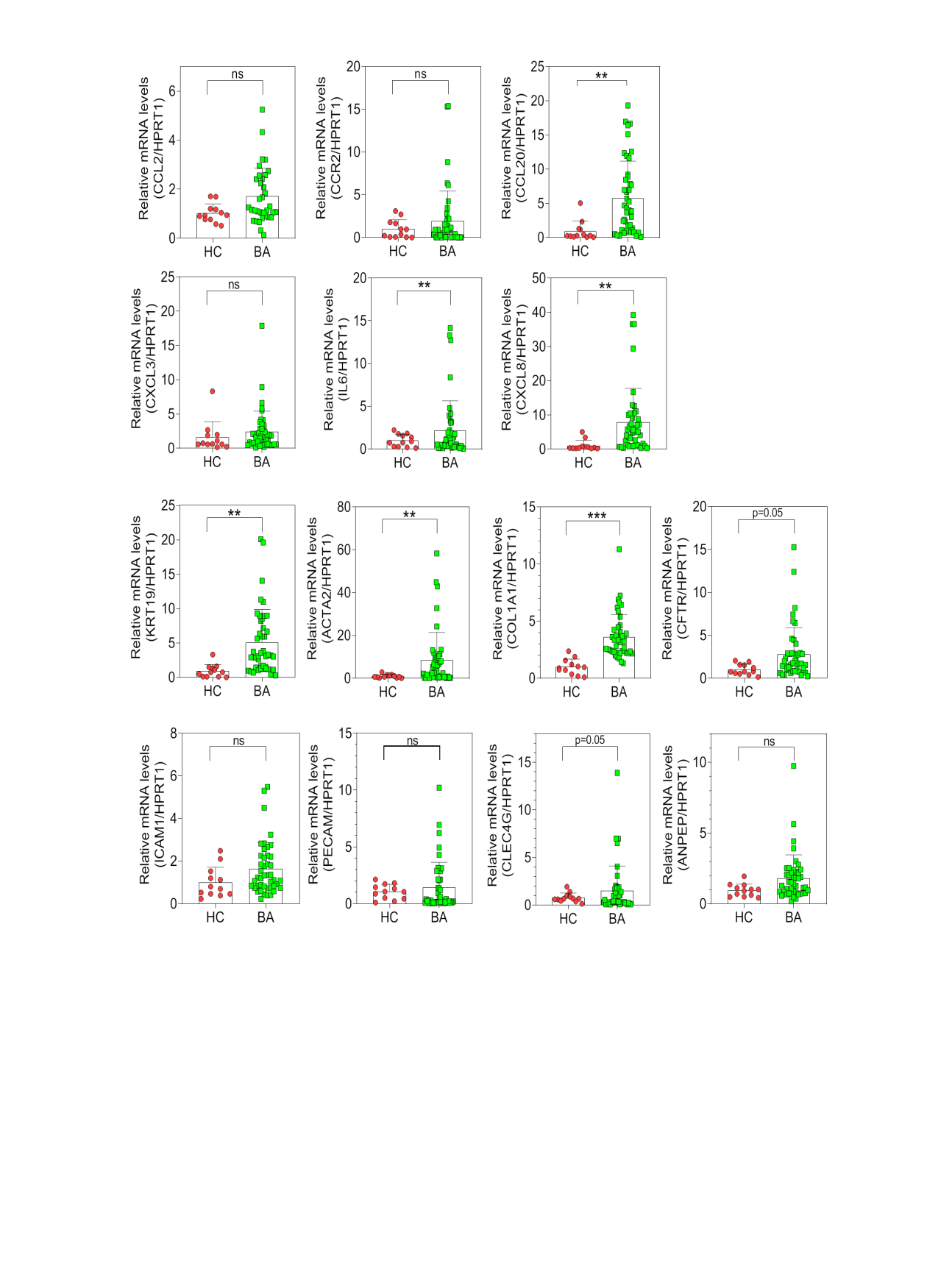
**

**Fig.S6. Hepatic macrophage gene levels correlated with the cholestatic liver injury, liver fibrosis and angiogenesis in BA patients.** The linear regression analysis showed that relative mRNA levels of hepatic CD11B were significantly correlated with the relative mRNA levels of CD68, CCL2, CCR2 and NOS2. The hepatic CD11B mRNA levels were correlated with levels of proinflammatory markers, including IL1B, TNFA and IL6. The figure indicates that hepatic CD11B expression levels were significantly correlated with the expression levels of KRT19 and fibrotic marker genes, including ACTA2, COL1A1 and CFTR. Furthermore, linear regression analysis showed that hepatic CD11B expression levels were correlated with the expression levels of angiogenesis marker genes, including ICAM1, PECAM, CLEC4G and ANPEP.

**
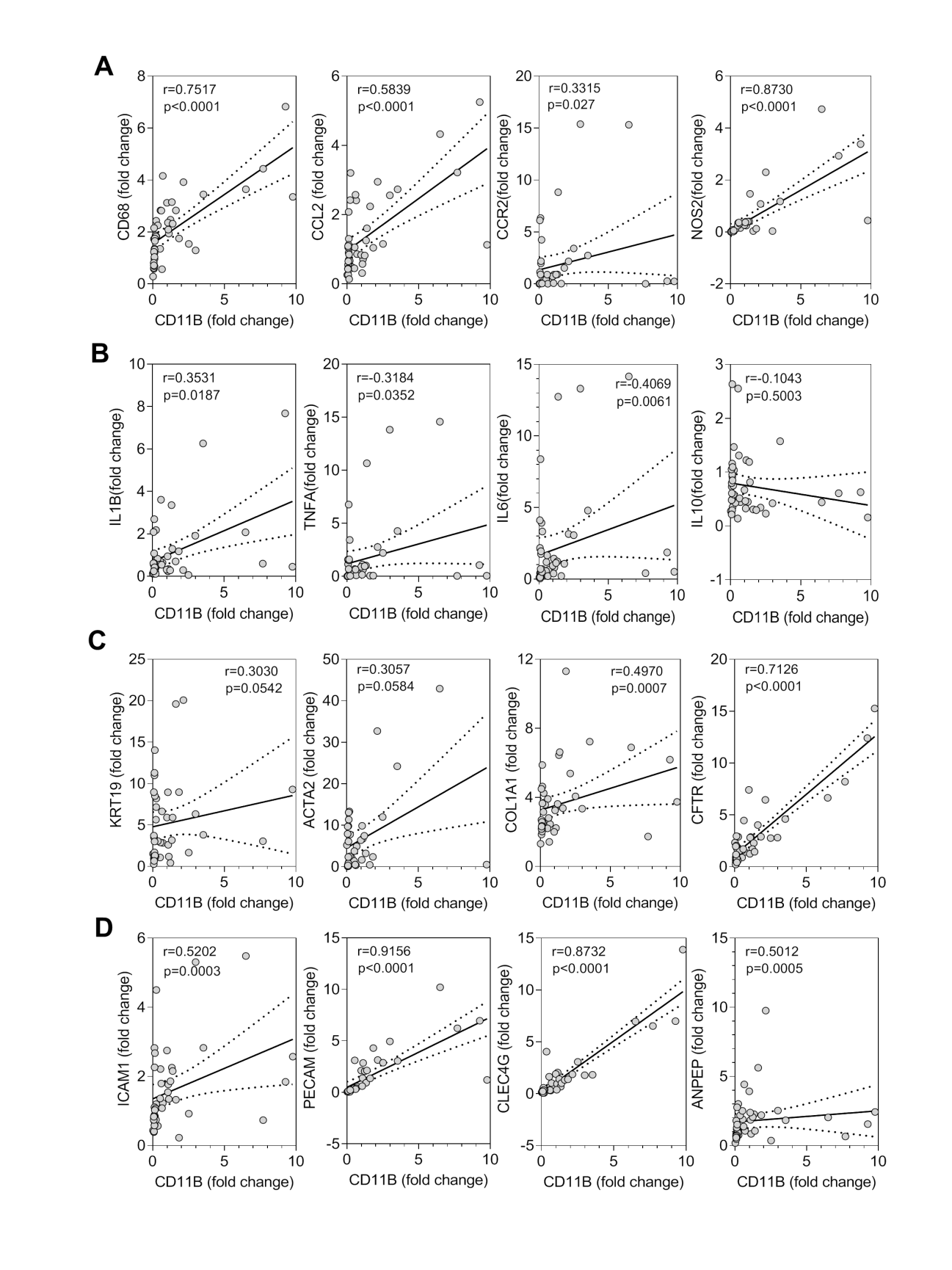
**

**Fig.S7. Diphtheria toxin (DT) injection depleted macrophages in the cholestatic livers of CD11b-DTR mice.**  (A) Quantification of Fig. 2C (B) The macrophage gene levels were detected in livers of Sham, Sham + DT, BDL and BDL+DT mice by RT-PCR assay. GAPDH was used as an internal control. Data were expressed as mean ± SD from five to eight mice per group. GAPDH, glyceraldehyde-3-phosphate dehydrogenase. *P < 0.05; **P<0.01, ***P < 0.001; ns, not significant.


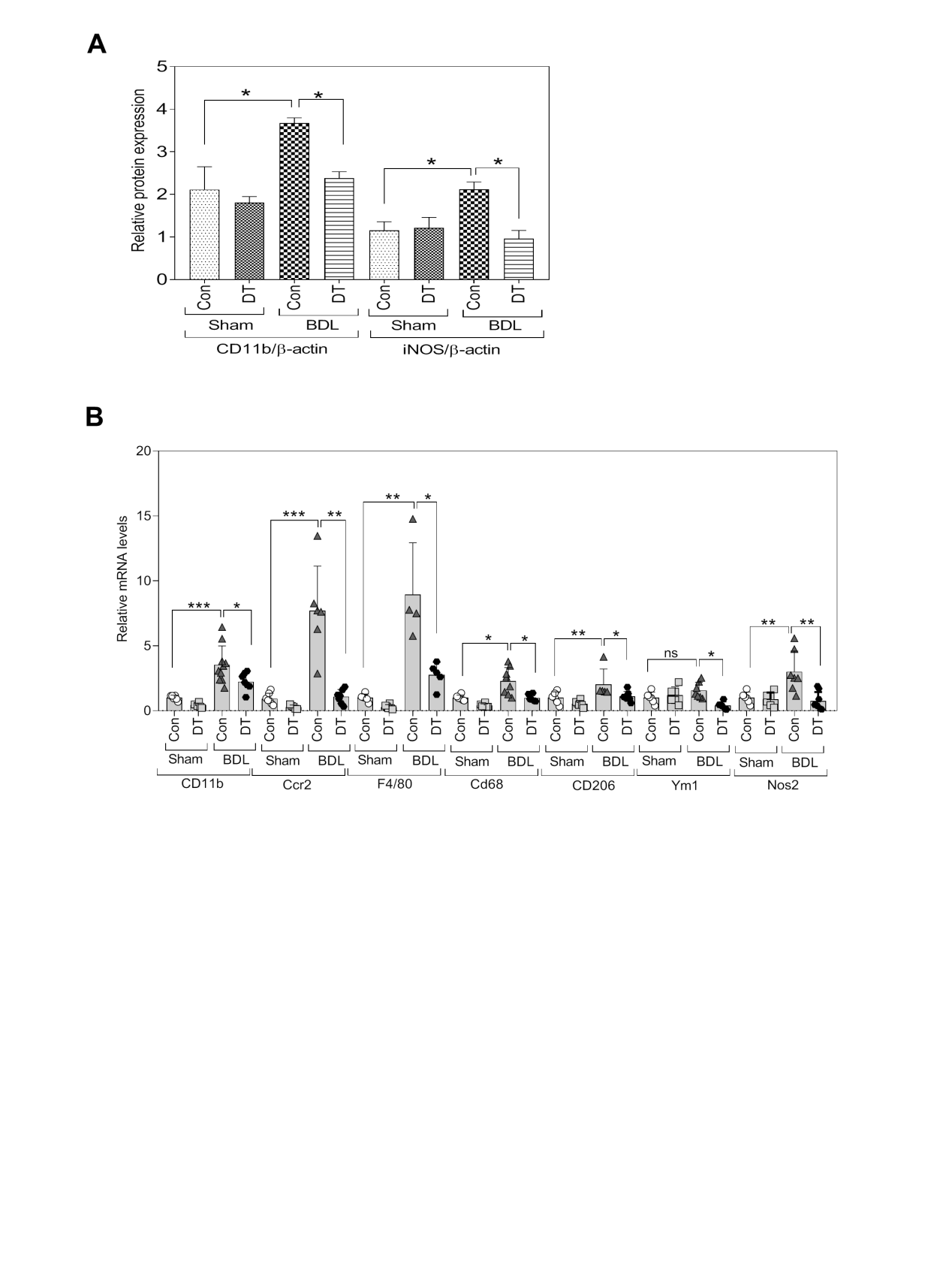


**Fig.S8. Diphtheria toxin (DT) injection did not deplete the macrophages in cholestatic livers of wild type mice.** (A) Representative images of immunofluorescence (IF) staining for CD11b, Ccr2, F4/80 and CD31 in the liver sections from BDL- WT and BDL- WT+DT mice. (B) Quantification of panel A. ns, not significant

**
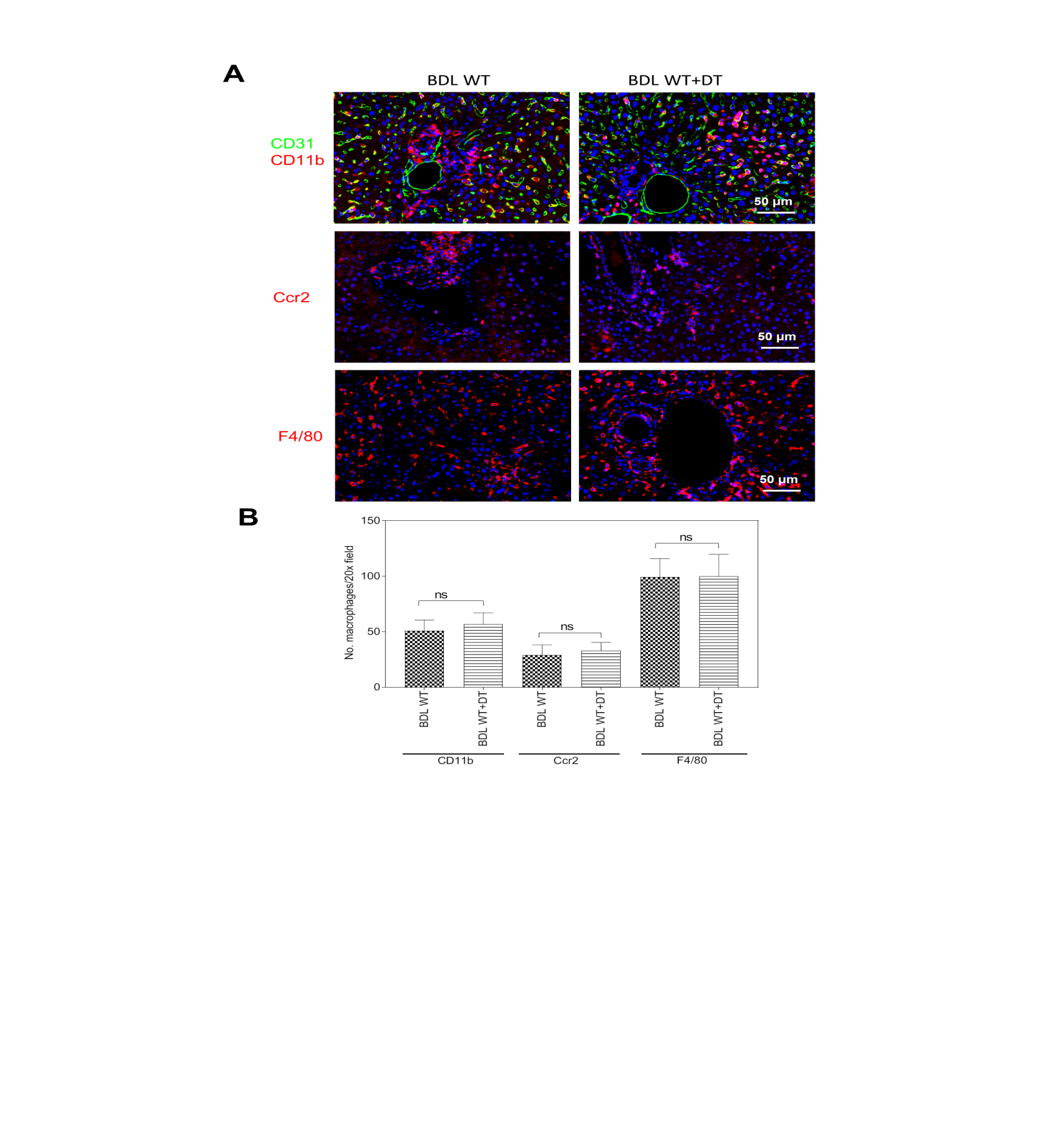
**

**Fig.S9. Macrophages depletion ameliorated BDL-induced liver fibrosis.** (A) Representative images of costaining of α-SMA and CCR2 in livers of Sham, Sham + DT, BDL and BDL+DT mice. (B) Quantification of perfibrotic nicho Ccr2^+^ cells in panel A. (C) Relative mRNA levels of fibrotic genes from Sham, Sham + DT, BDL and BDL+DT mice (n = 5-8) were determined by RT-PCR and normalized using GAPDH as an internal control. (D) Hepatic hydroxyproline content was measured. Data were expressed as mean ± SD from five to eight mice per group. *P < 0.05; **P<0.01; ***P<0.001

**
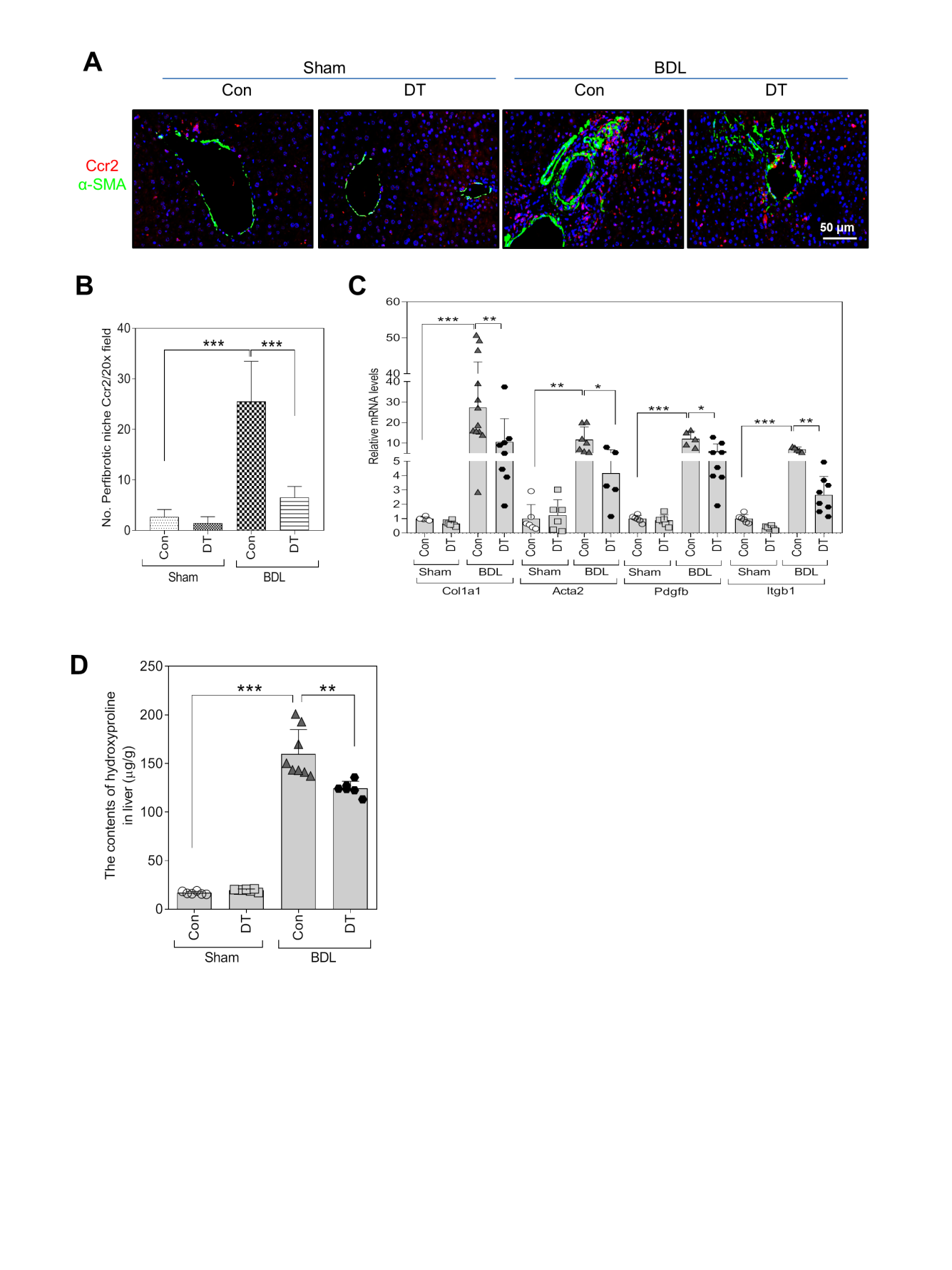
**

**Fig.S10. Macrophage depletion inhibited BDL-induced cholangiocyte proliferation in liver.** (A) Representative images of costaining of CK19 and F4/80 in livers of Sham, Sham + DT, BDL and BDL+DT mice (n = 5-8). (B) Quantification of panel A. (C) Relative mRNA levels of the cholangiocytes’ markers and inflammatory genes from Sham, Sham + DT, BDL and BDL+DT mice (n = 5-8) were determined by RT-PCR and normalized using GAPDH as an internal control. Data were expressed as mean ± SD. *P < 0.05; **P<0.01; ***P<0.001; ns, not significant


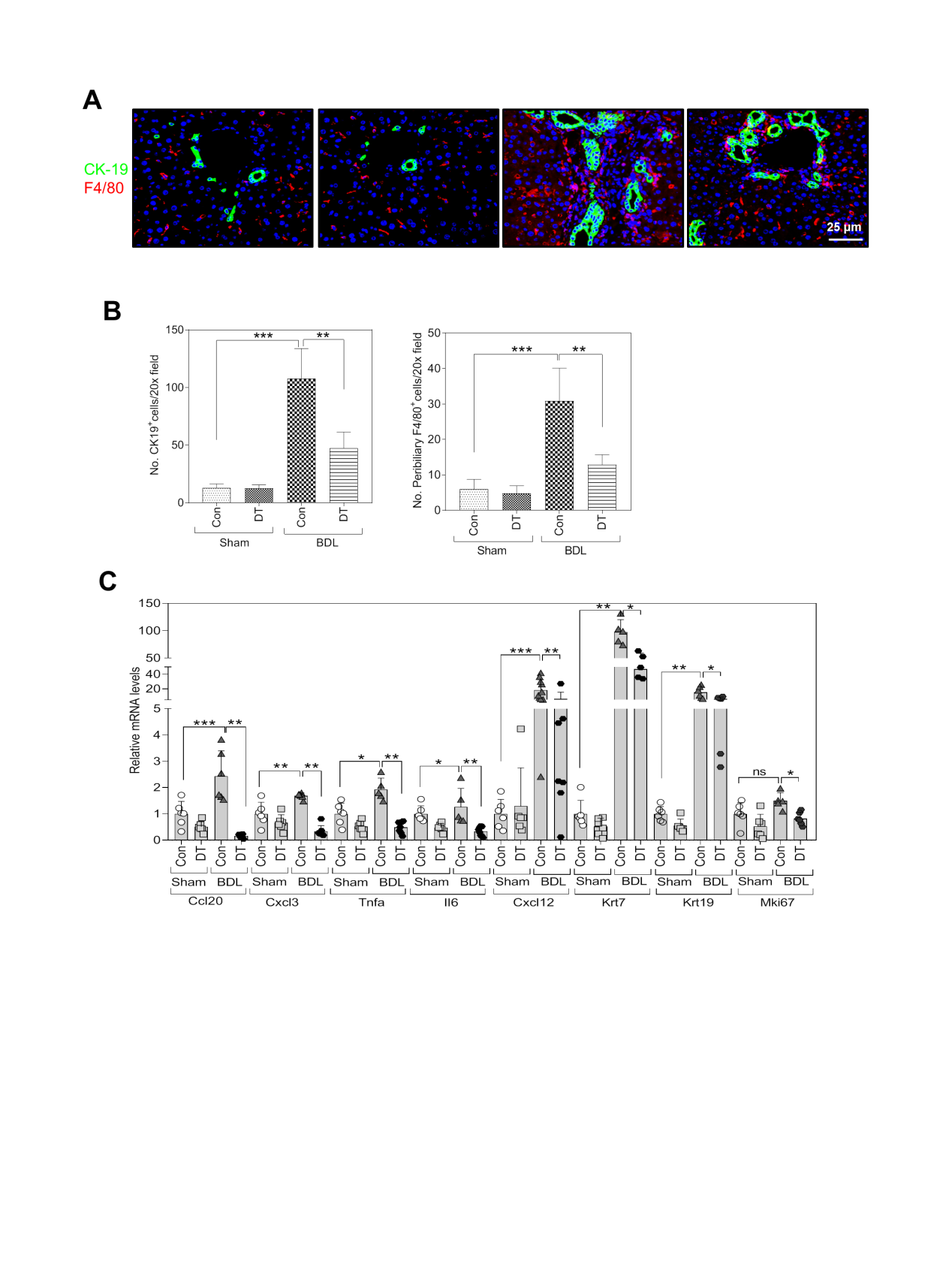


**Fig.S11. Macrophage depletion inhibited BDL-induced angiogenesis in liver.** (A) The angiogenesis gene levels were detected in livers of Sham, Sham+DT, BDL and BDL+DT mice by RT-PCR assay. GAPDH was used as an internal control. GAPDH, glyceraldehyde-3-phosphate dehydrogenase; (B) Immunofluorescence (IF) staining images of CD31 in Sham, Sham+DT, BDL and BDL+DT mice. (C) Quantification of panel B. Data were expressed as mean ± SD from five to eight mice per group. *P < 0.05; **P<0.01; ***P<0.001; ns, not significant.


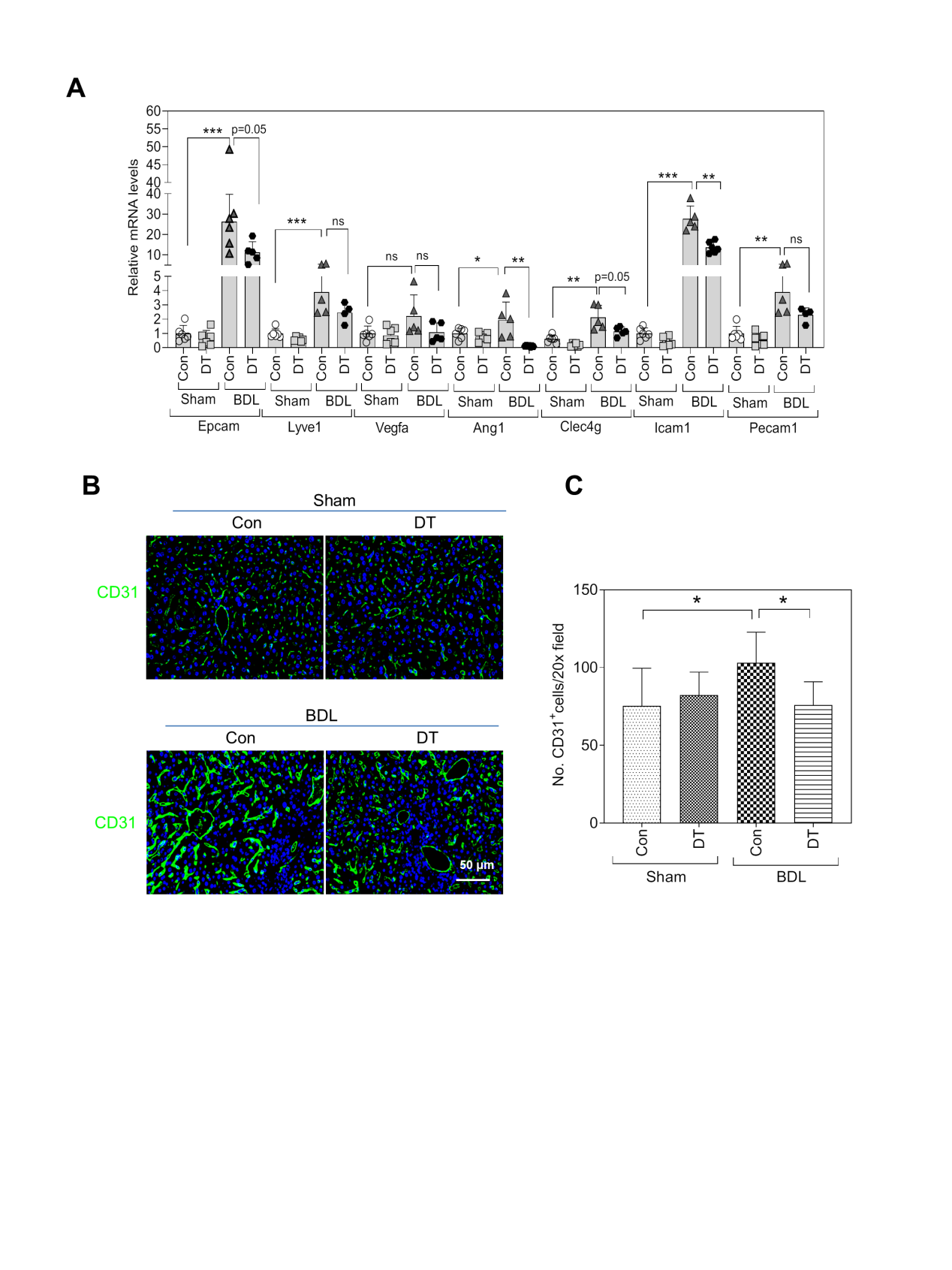


**Fig.S12. H19 knockout reduced the BDL-induced cholangiocyte proliferation and liver fibrosis.** (A) Representative images of Masson’s trichrome staining, scale bars: 500 μm, and Sirius red staining, scale bars: 50 μm for the livers sections from Wt sham, H19^-/-^ Sham, Wt BDL and H19^-/-^ BDL mice (Each group, n=3-6). (B, C) Quantification of Masson area and Sirius area in panel A. (D-G) Western blotting analysis for CK19, Pcna, Collagen I, α-SMA, β-tubulin and β-actin in livers of Wt sham, H19^-/-^ Sham, Wt BDL and H19^-/-^ BDL mice. Representative image of the immune blotting and quantification of all of them are shown. Quantification of panel B. Data were expressed as mean ± SD. *P < 0.05; **P<0.01, ***P<0.001; ns, not significant.

**
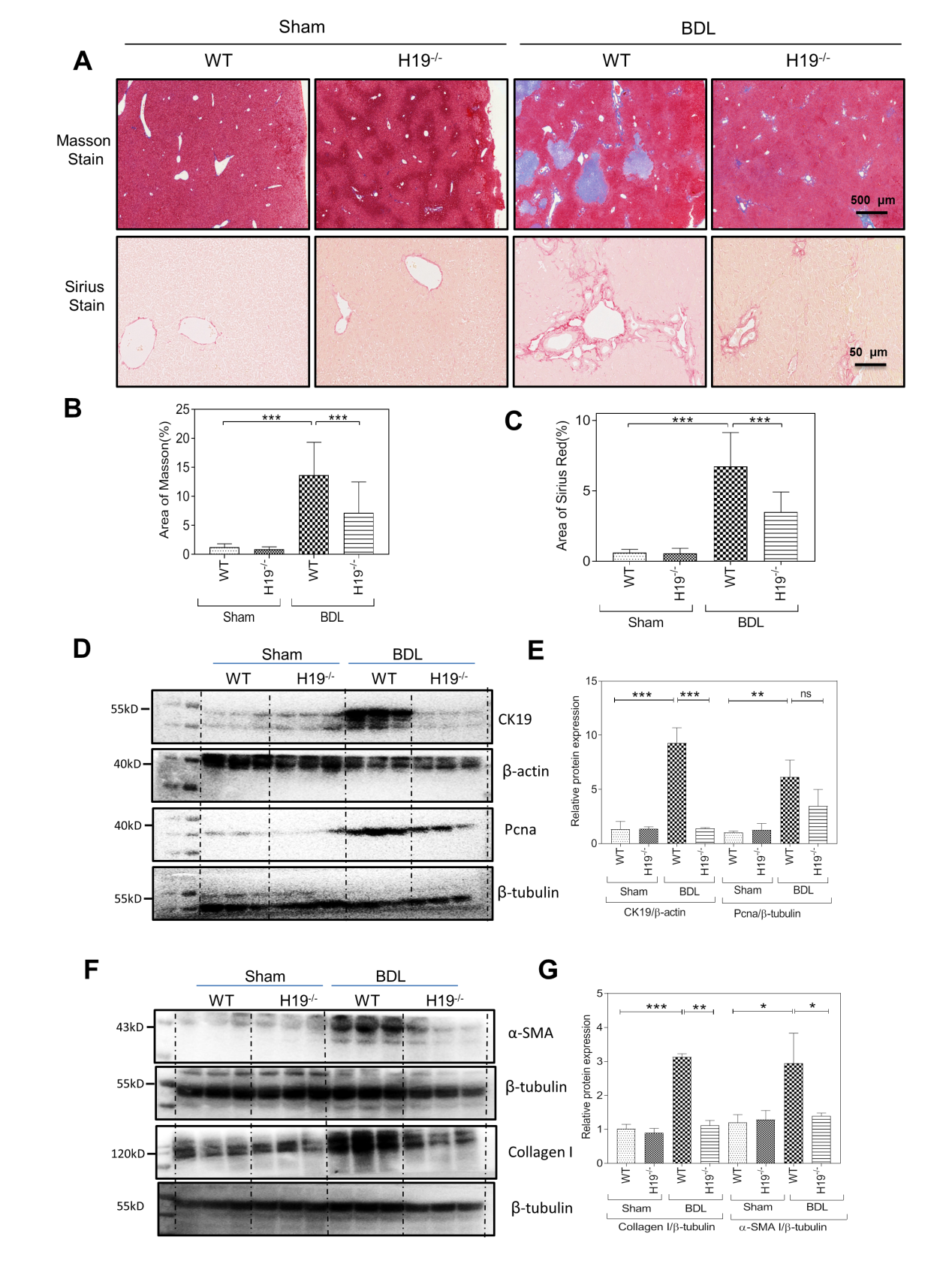
**

**Fig.S13. H19 overexpressed in the livers of mice using adenovirus vector.** (A, B) A dose of 1.0 × 10^11^ vector genomes of the AAV9-H19 or controls were injected into CD11b-DTR mice via tail vein. After one-week of injection, the mice were subjected to the BDL operation for 10 days and randomly divided into 4 groups: AAV9-CTL, AAV9-H19, AAV9-CTL+DT and AV9-H19+DT (each group, n = 4 - 6). Hepatic levels of H19 were determined by RT-PCR. Data were expressed as mean ± SD. *P < 0.05; **P<0.01; ns, not significant.

**
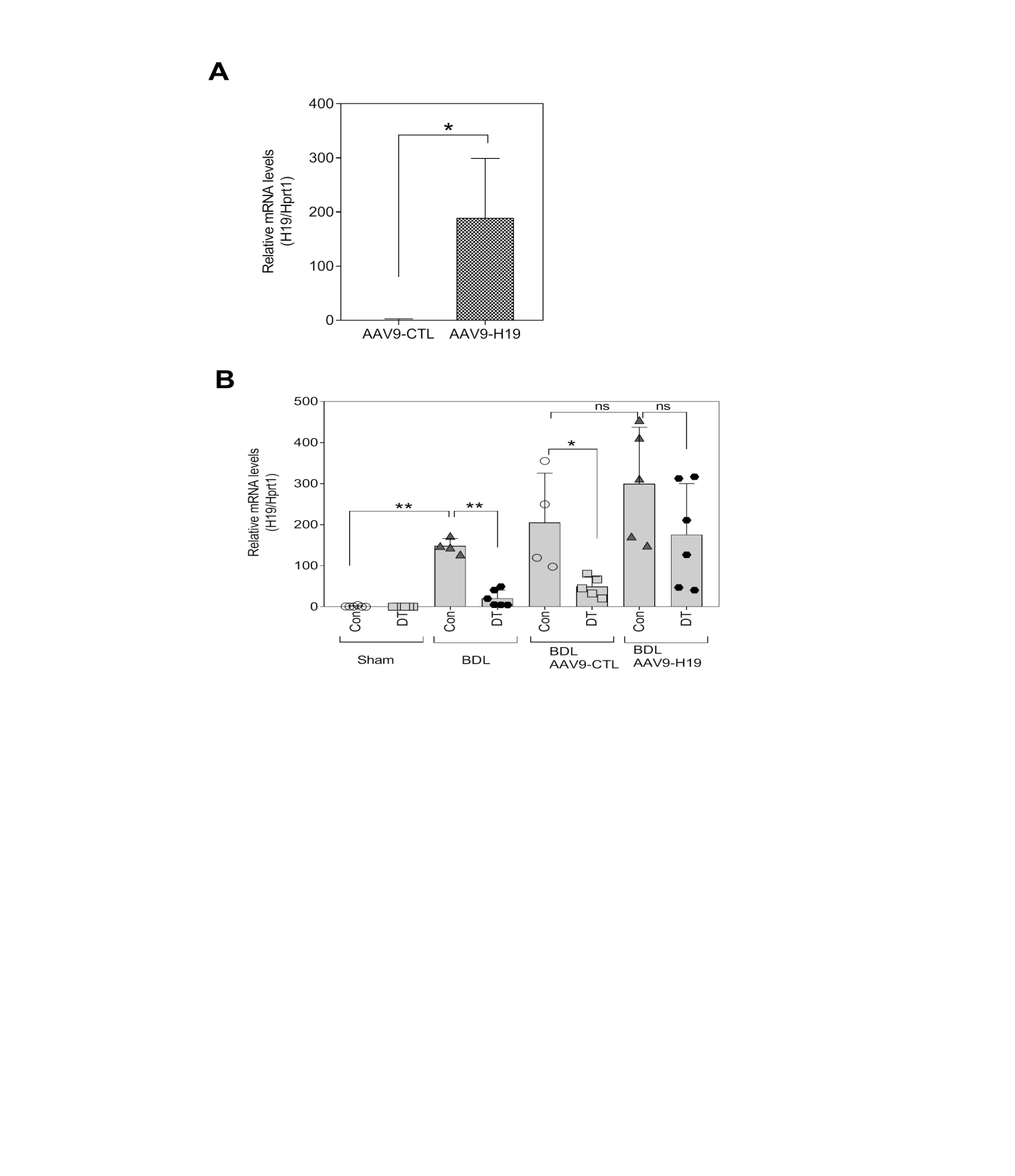
**

**Fig.S14. Tamoxifen (TAM) treatment depleted H19 in macrophages.** (A) Representative images of H19 Fluorescence *in situ* hybridization (FISH) and CD11b immunofluorescence (IF) co-staining in liver sections from BDL and BDL+ Tamoxifen mice. Scale bars: 25 μm.


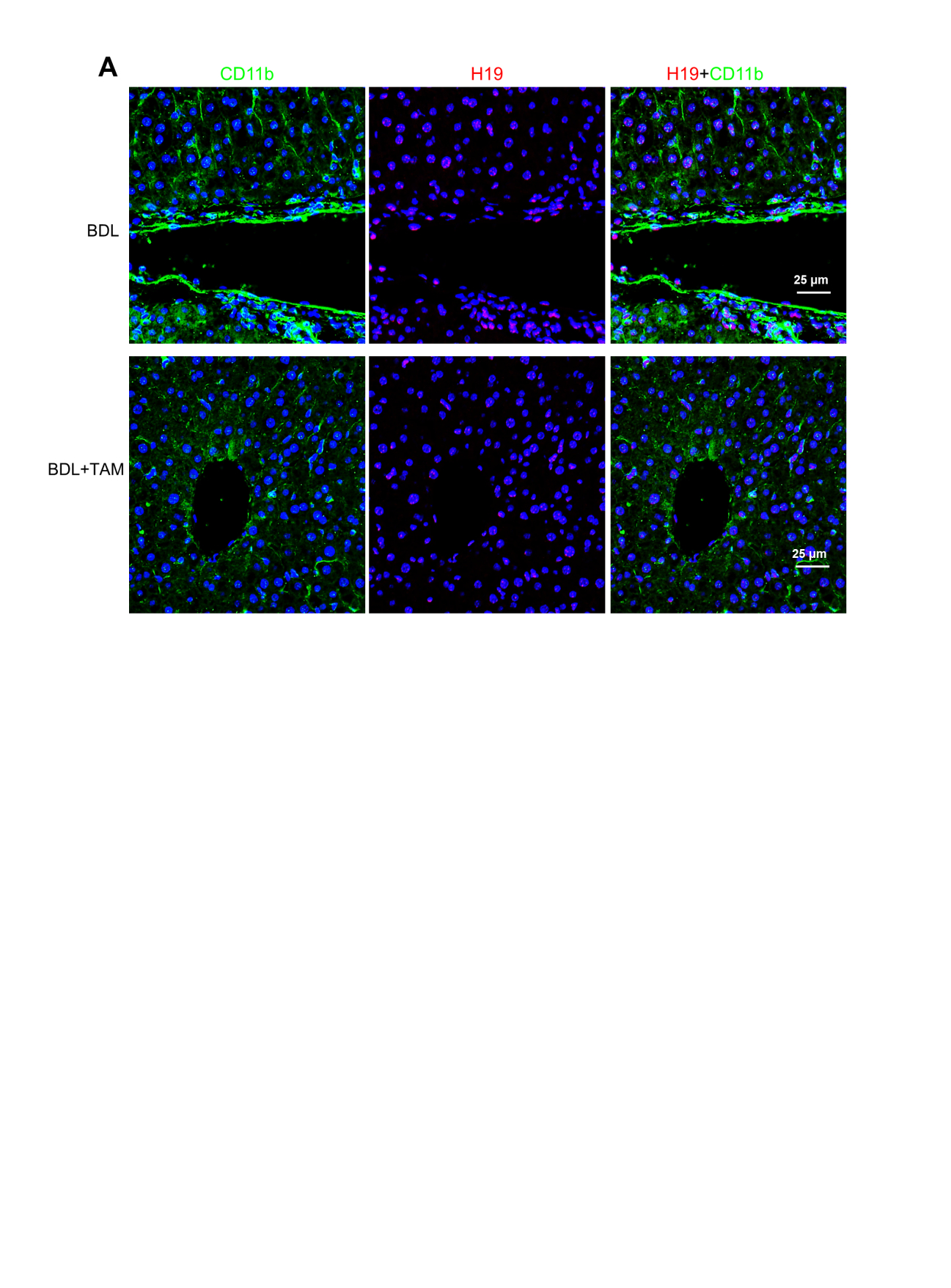


**Fig.S15. H19 knockout depressed the activation and polarization of macrophages in the cholestatic livers.** (A) Representative images of co-staining of CD31 and CD11b, α-SMA and Ccr2, F4/80 and CK19 in the liver sections from Wt sham, H19^-/-^ Sham, Wt BDL and H19^-/-^ BDL mice. (Each group, n=3-6). (B) Quantification of panel A. Data were expressed as mean ± SD. **P<0.01, ***P < 0.001

**
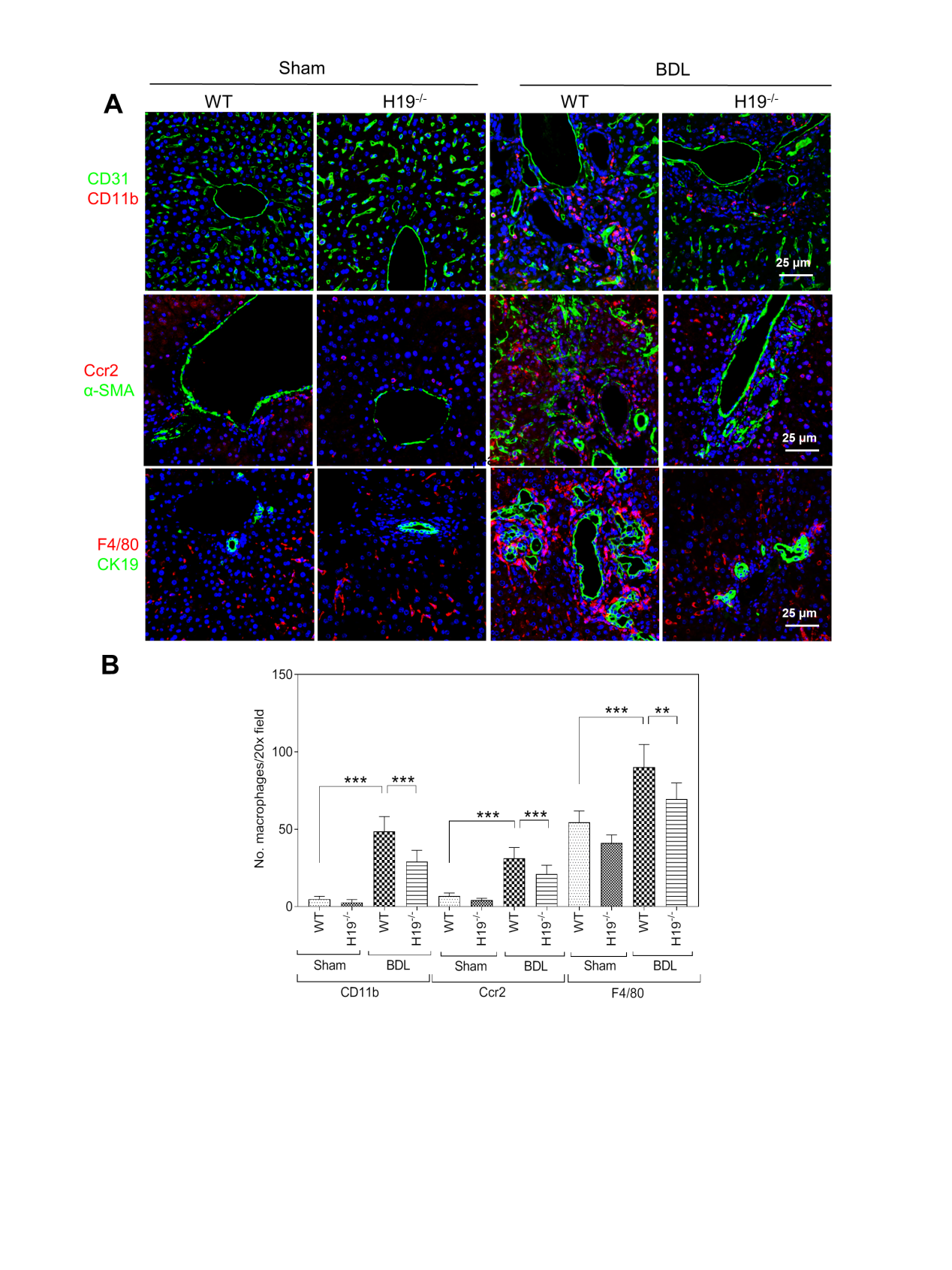
**

**Fig.S16. H19 knockout repressed proteins of macrophages in the cholestatic livers.** (A) Western blot analysis for CD11b, iNos, Ccr2 and β-actin in the liver tissues from Wt sham, H19^-/-^ Sham, Wt BDL and H19^-/-^ BDL mice. (B) Quantification of panel A. Data were expressed as mean ± SD. *P < 0.05; **P<0.01; ***P < 0.001


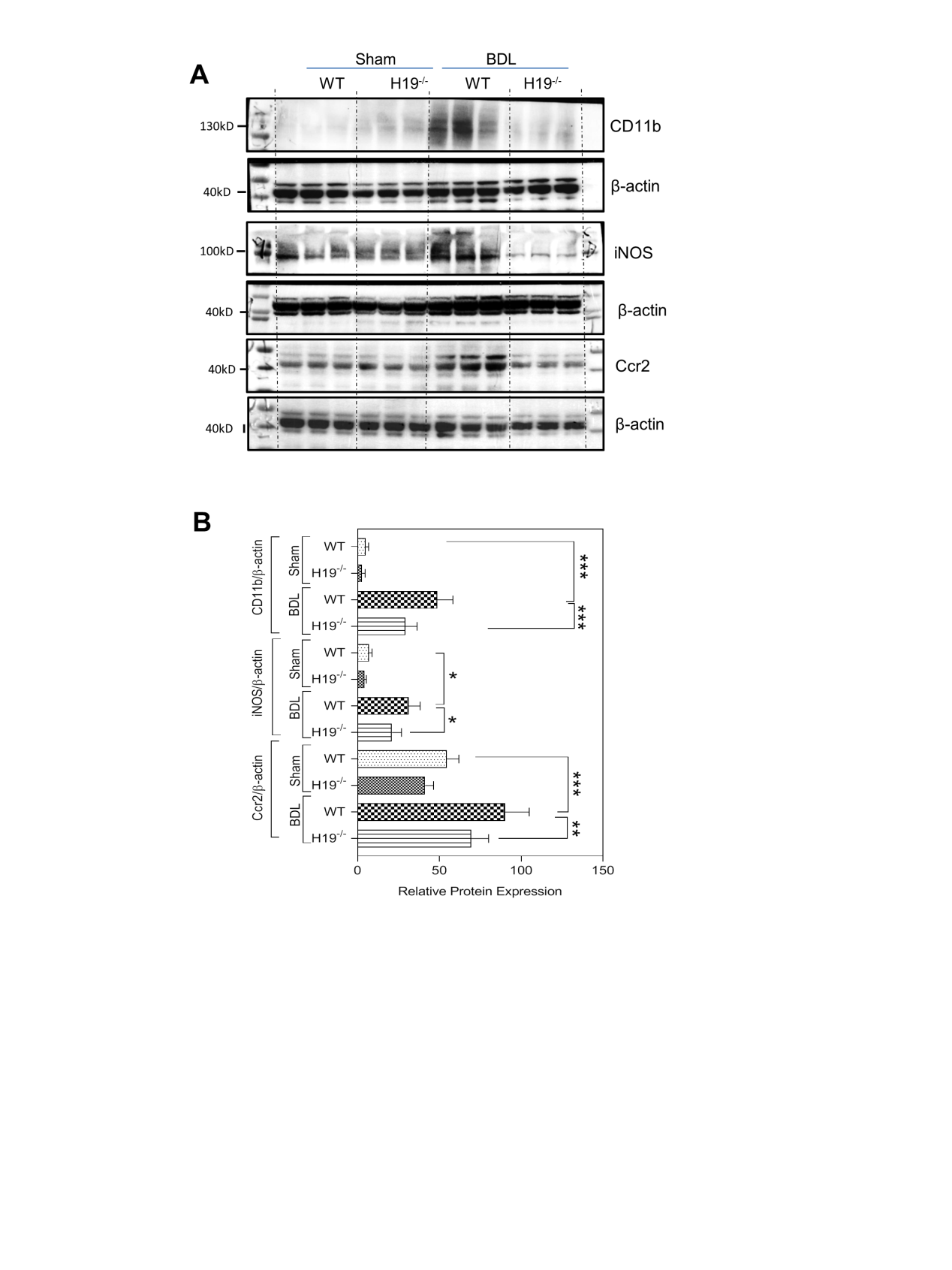


**Fig.S17. H19 knockout reduced monocytes in BDL mice.** (A) Representative flow cytometry results and images of the percentage of indicated cells in all isolated cells are shown. (B) Quantification of panel A. Data were expressed as mean ± SD. **P<0.01; ns, not significant.

**
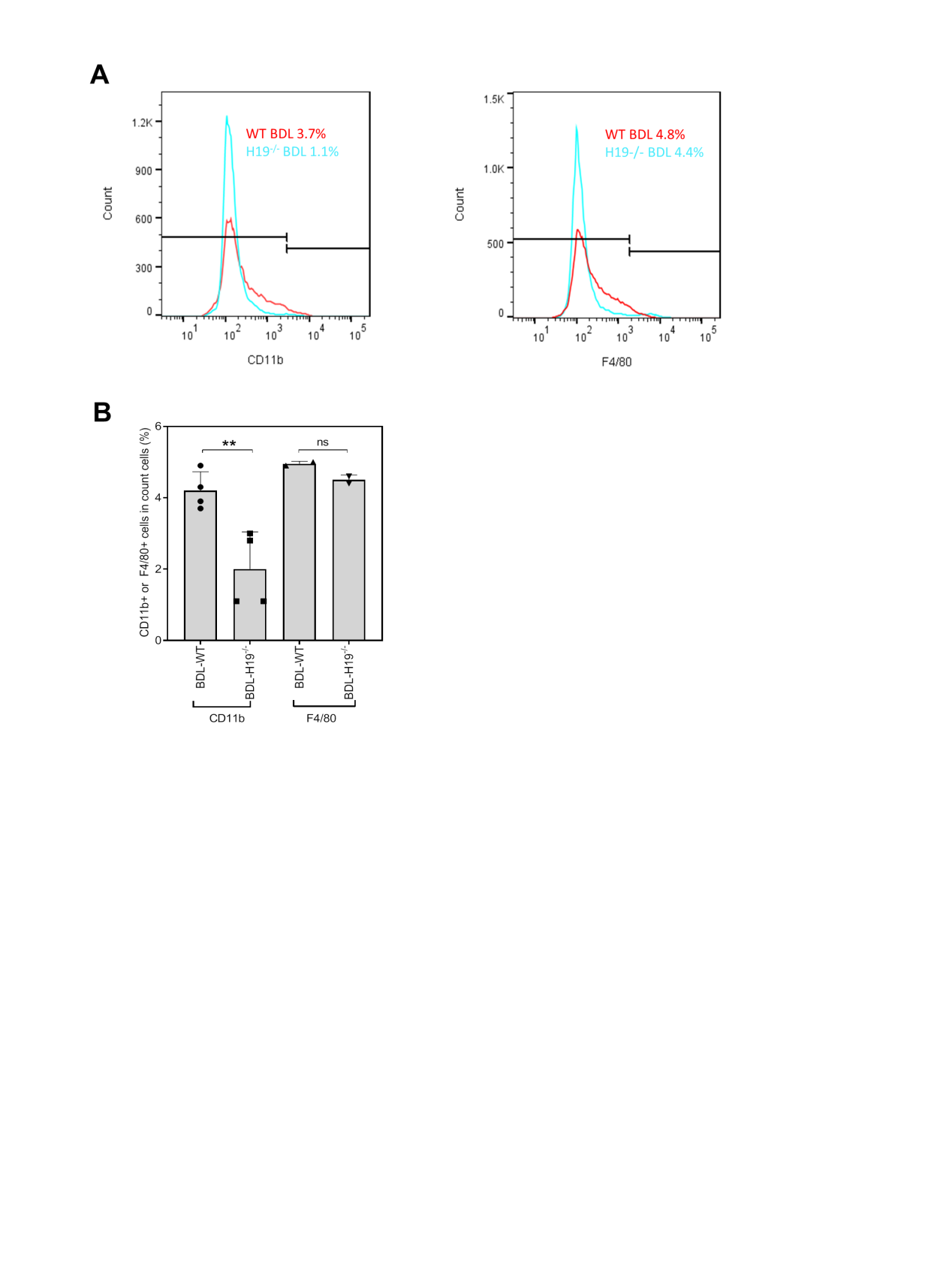
**


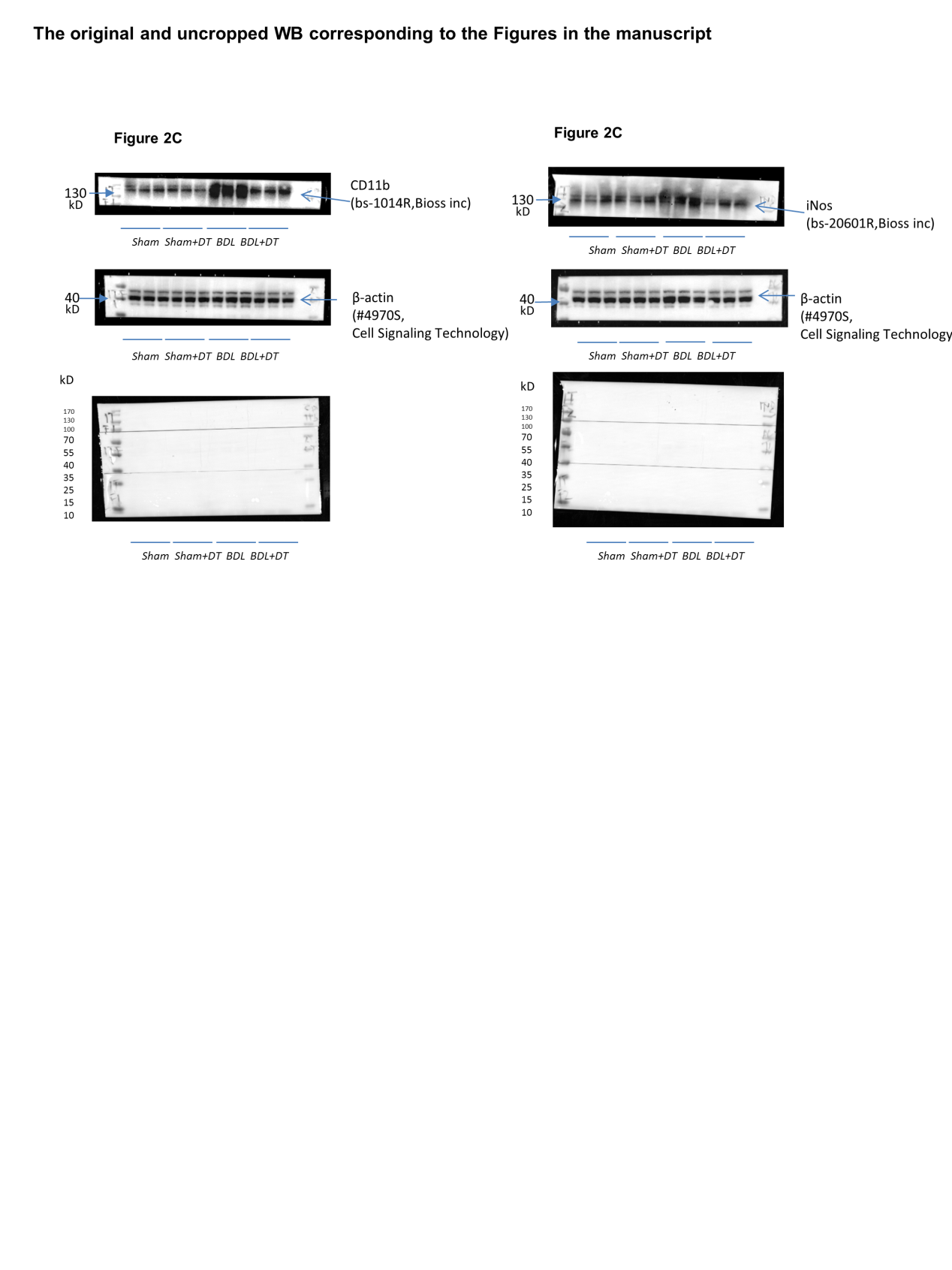


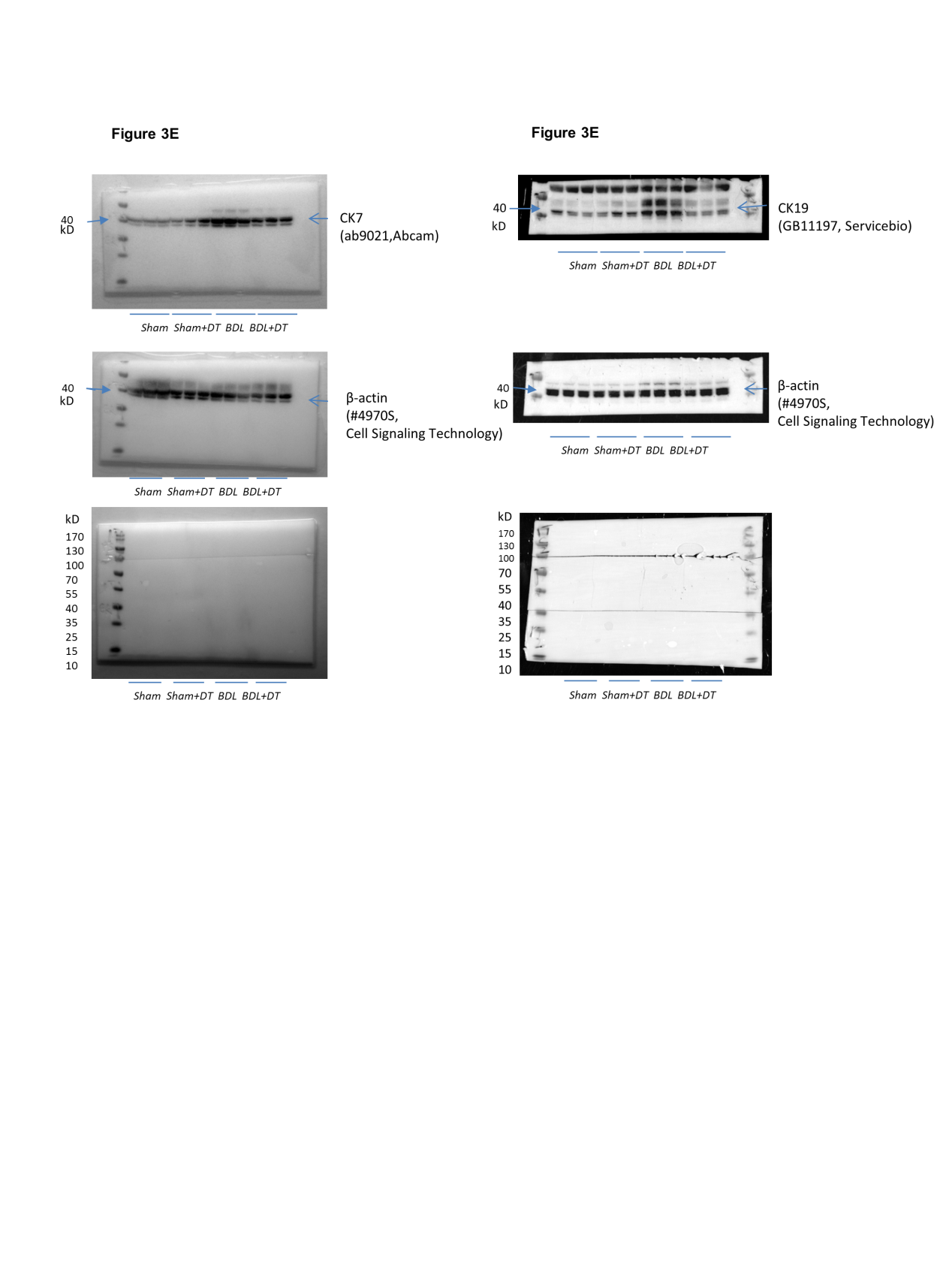


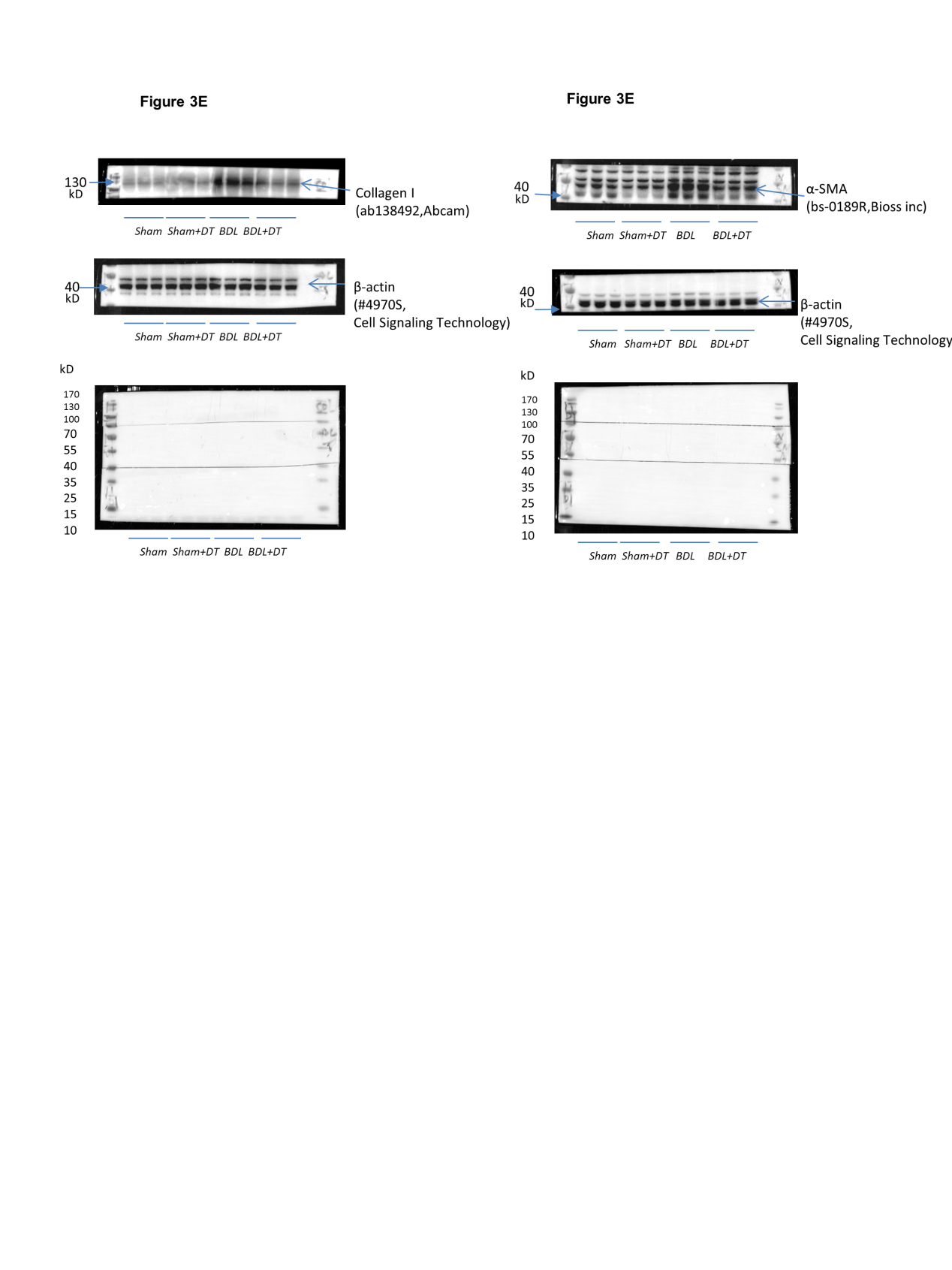


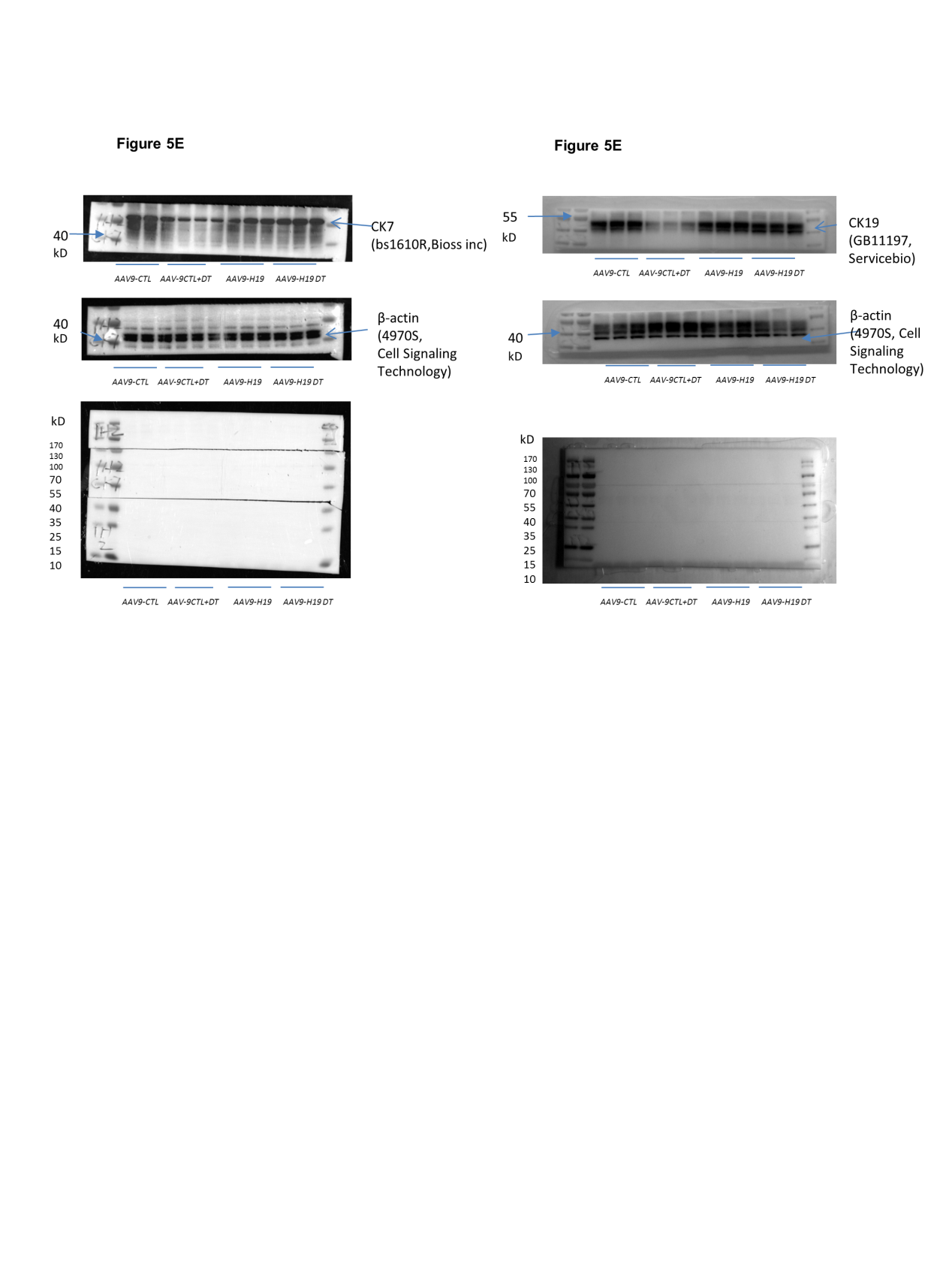


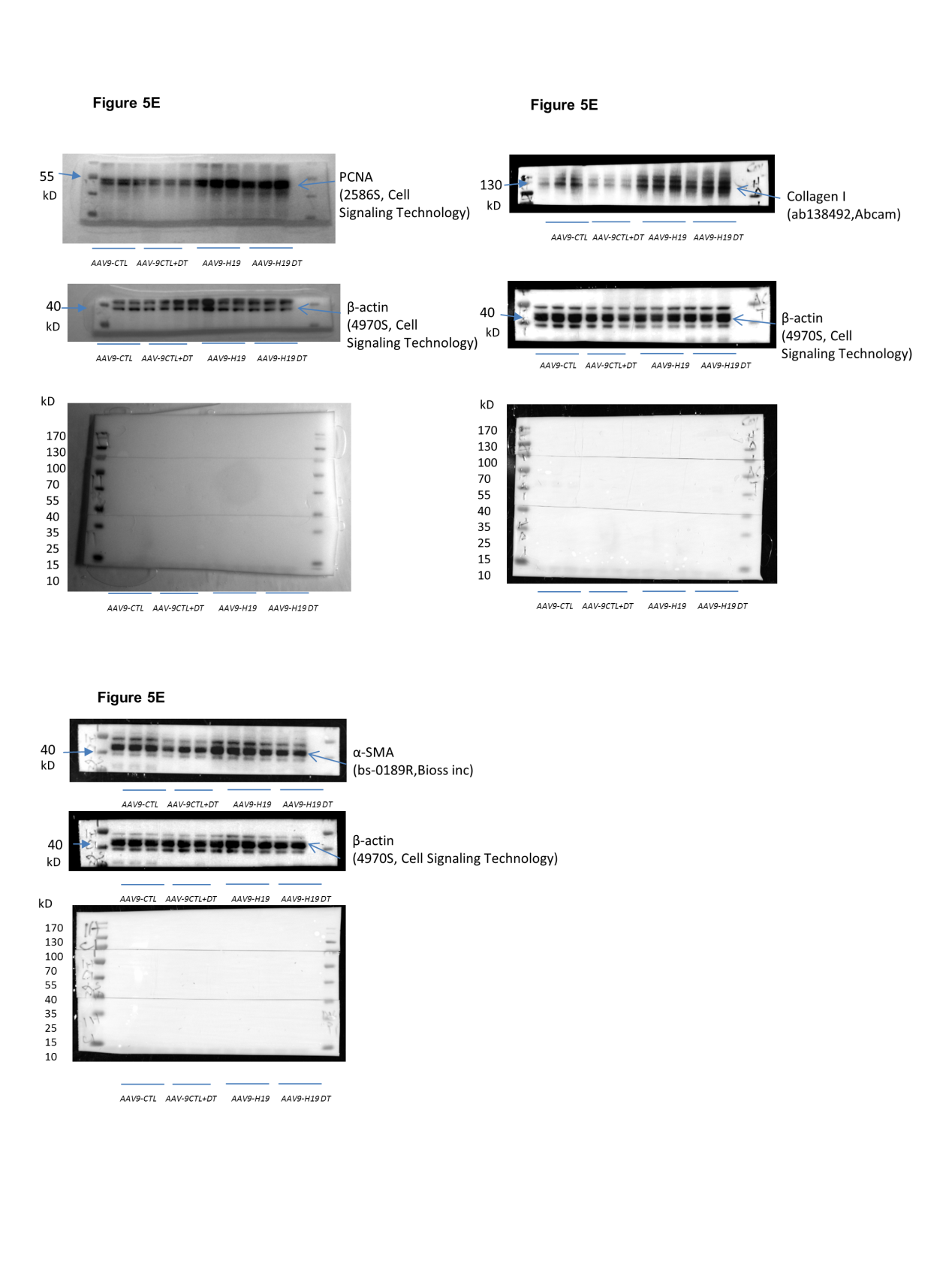


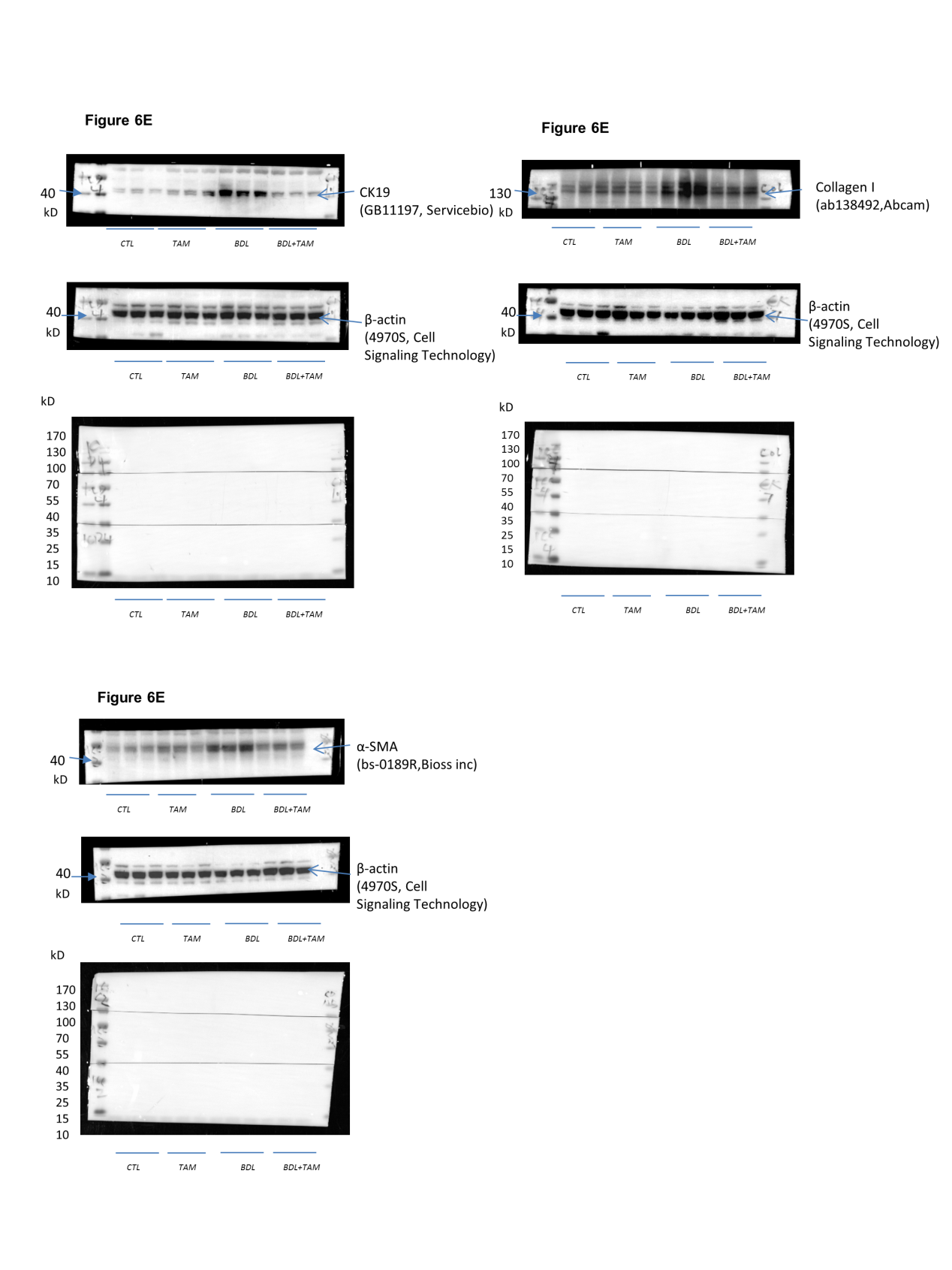


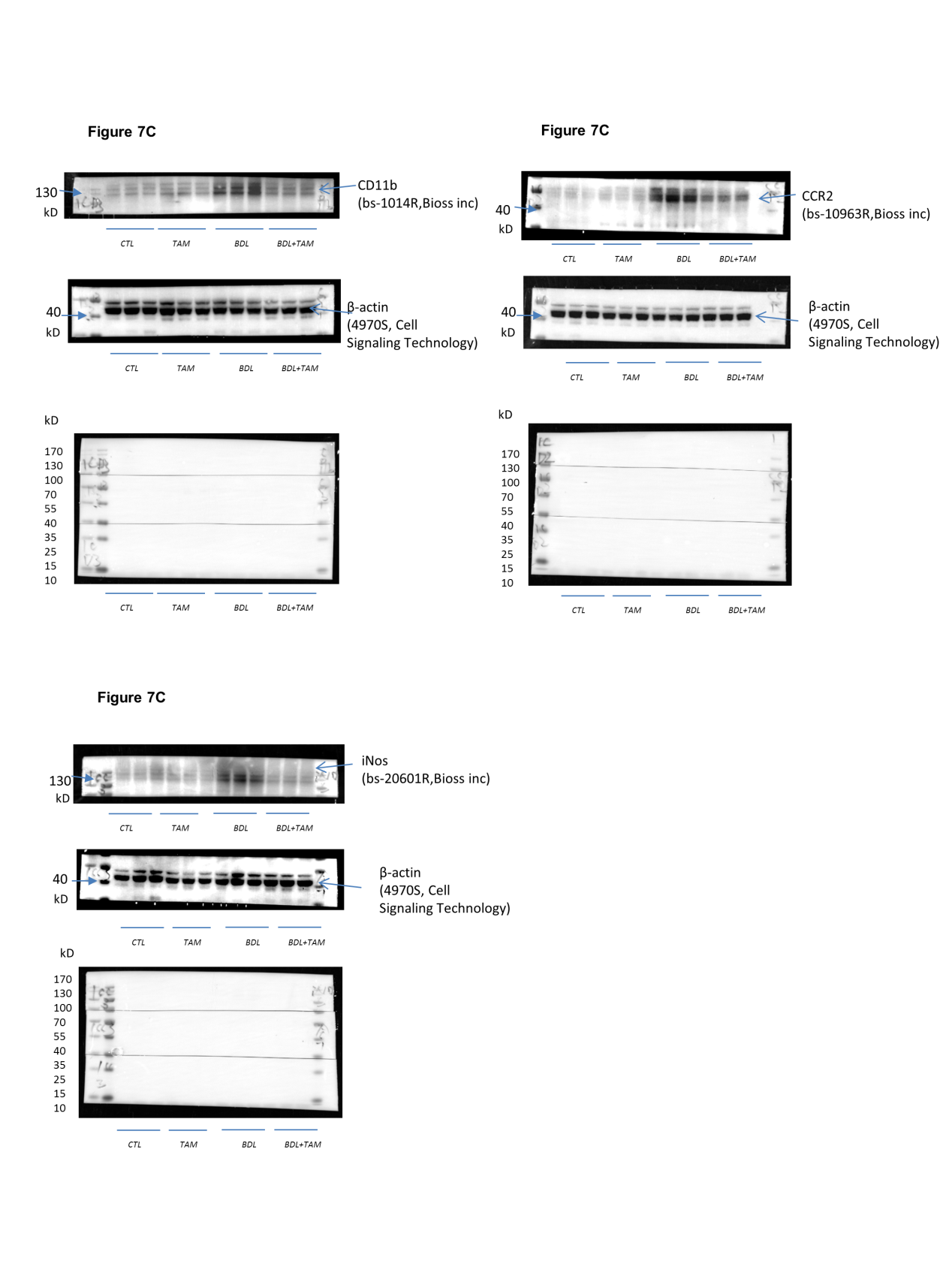


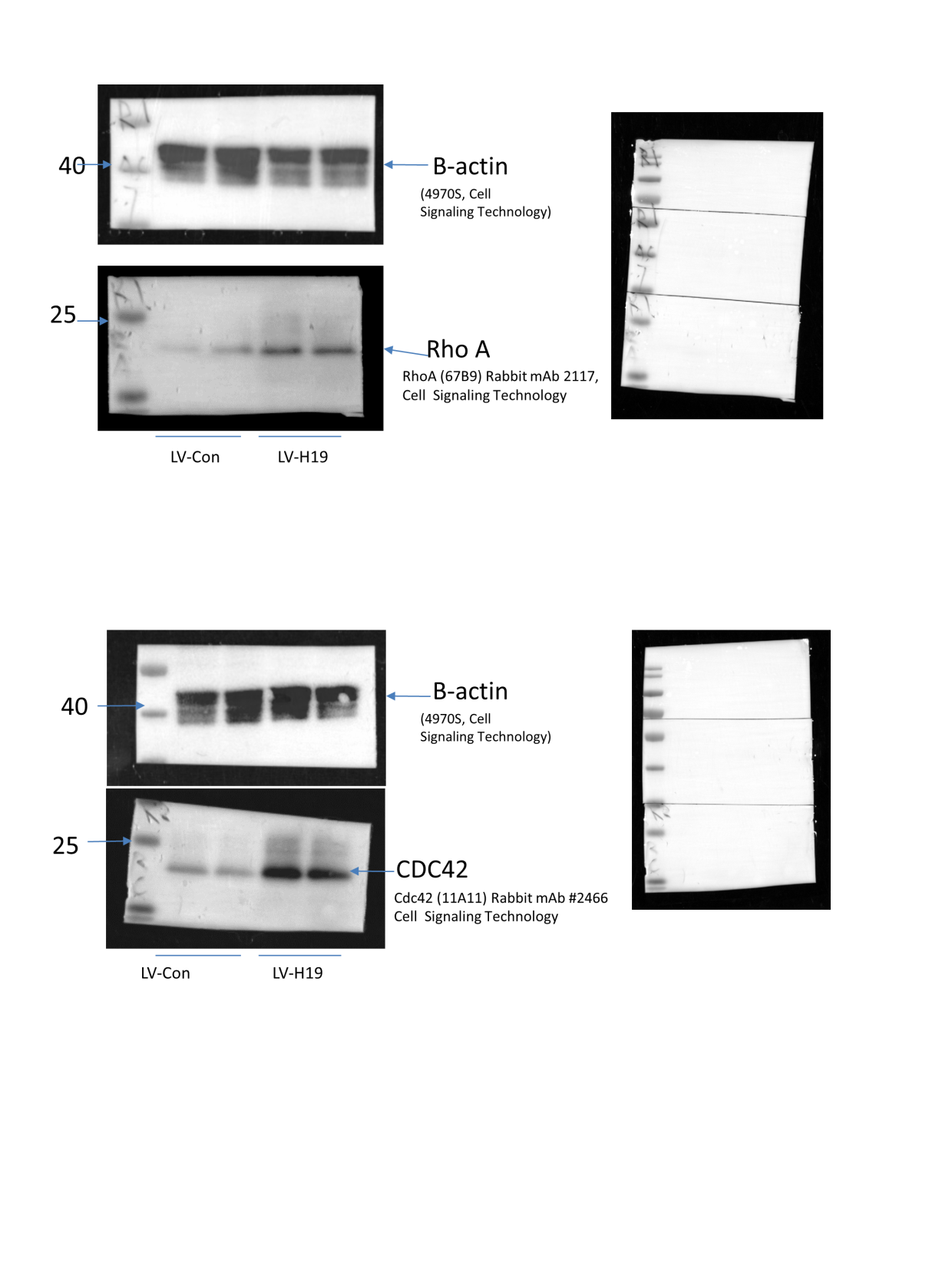
 **Figure 8D**


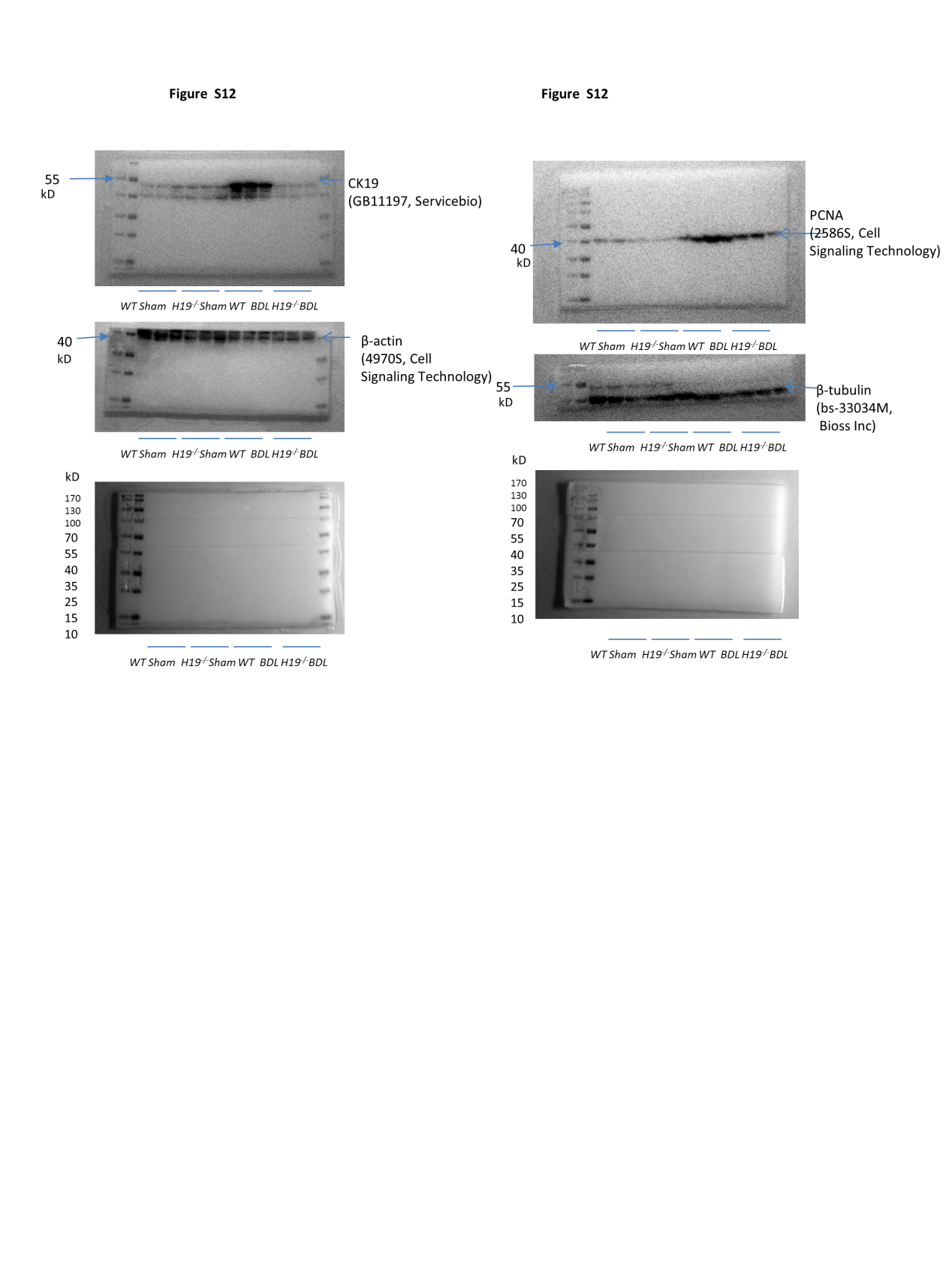


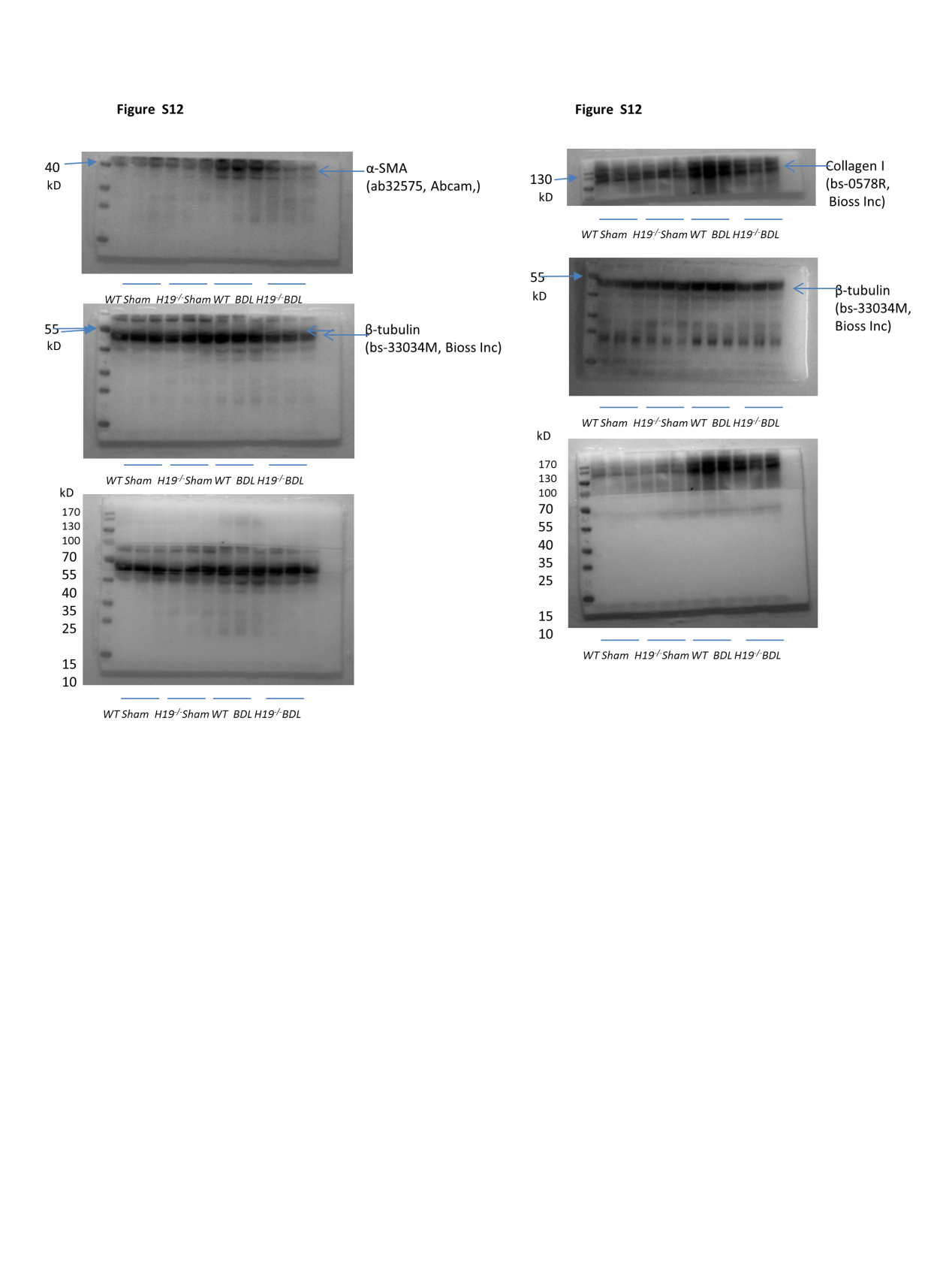


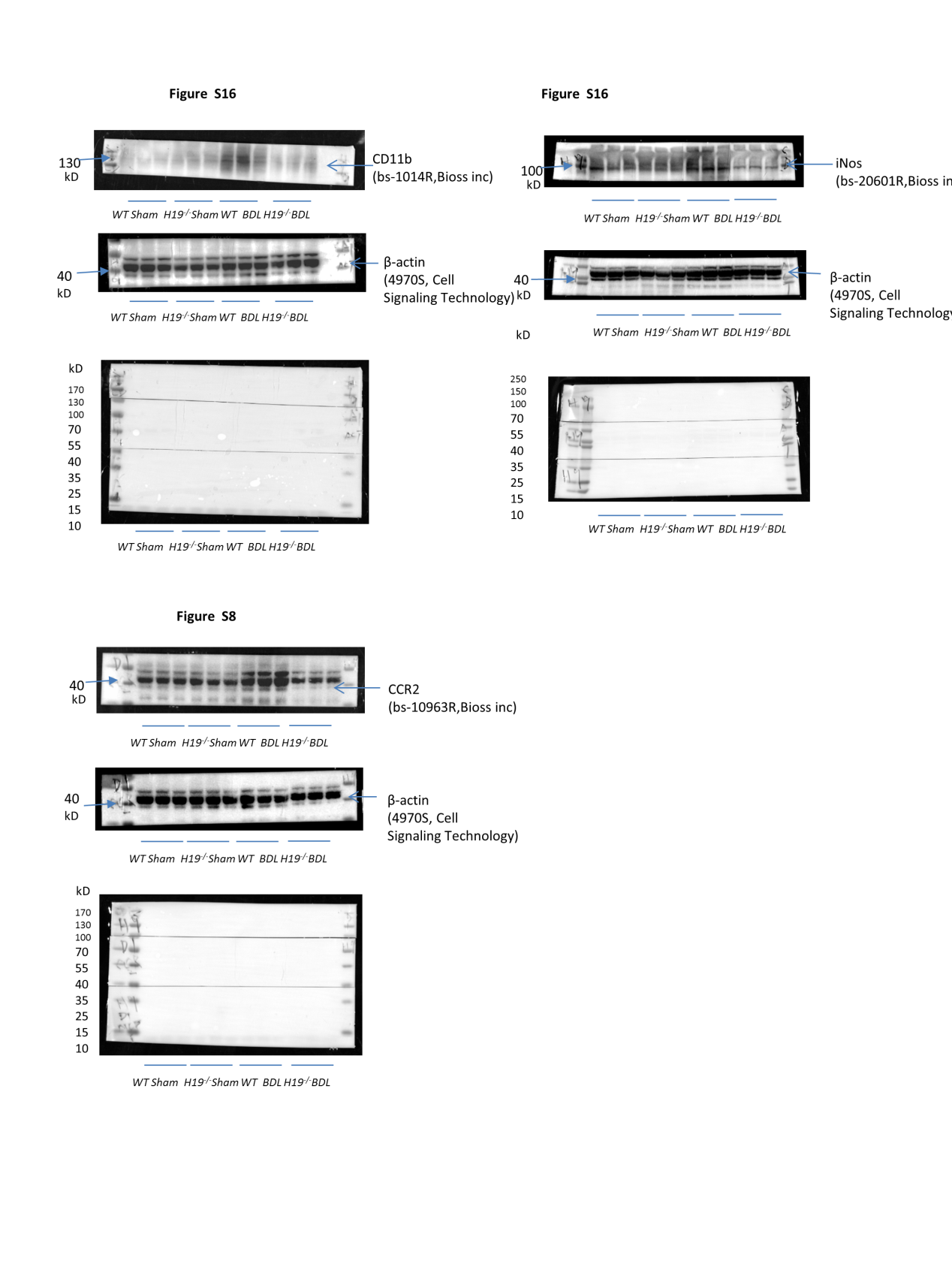

Supplement: Supplementary file 1 — CDDIS-21-1265R-revised Supplemental with clean version [file 41419_2021_3931_MOESM1_ESM.docx]
